# Posttranscriptional Control of Neural Progenitors Temporal Dynamics During Neocortical Development by Syncrip

**DOI:** 10.1002/advs.202411732

**Published:** 2025-01-07

**Authors:** Jiarui Wu, Haoyang Yu, Xinyi Dou, Bin Yin, Lin Hou, Yuanchao Xue, Boqin Qiang, Pengcheng Shu, Xiaozhong Peng

**Affiliations:** ^1^ State Key Laboratory of Common Mechanism Research for Major Diseases Department of Biochemistry & Molecular Biology Medical Primate Research Center Neuroscience Center Institute of Basic Medical Sciences Chinese Academy of Medical Sciences School of Basic Medicine Peking Union Medical College Beijing 100005 China; ^2^ Key Laboratory of RNA Biology Institute of Biophysics Chinese Academy of Sciences Beijing 100101 China; ^3^ Chinese Institute for Brain Research Beijing 102206 China; ^4^ State Key Laboratory of Respiratory Health and Multimorbidity Institute of Laboratory Animal Science Chinese Academy of Medical Sciences & Peking Union Medical College Beijing 100021 China

**Keywords:** neocortex, neural progenitors, neurodevelopment disorders, phase separation, posttranscriptional regulation, syncrip

## Abstract

The development of the mammalian neocortex is precisely regulated by temporal gene expression, yet the temporal regulatory mechanisms of cortical neurogenesis, particularly how radial glial cells (RGCs) sequentially generate deep to superficial neurons, remain unclear. Here, the hnRNP family member Syncrip (hnRNP Q) is identified as a key modulator of superficial neuronal differentiation in neocortical neurogenesis. Syncrip knockout in RGCs disrupts differentiation and abnormal neuronal localization, ultimately resulting in superficial cortical layer defects as well as learning and memory impairments in mice. Single‐cell RNA sequencing analysis demonstrated that the knockout of Syncrip disrupts the late‐stage neurogenesis, stalling transcriptional progression in RGCs. Mechanistically, Syncrip maintains the transcription of temporal process‐related transcription factors by recruiting stabilization complexes through phase separation, crucially regulating the Notch signaling pathway that determines the fate of RGCs. Furthermore, pathogenic human mutations in Syncrip weaken its phase‐separation capability, failing to form stable complexes normally. Thus, Syncrip acts as a mediator of posttranscriptional regulatory mechanisms, governing the fate progression of RGCs and the advancement of intrinsic temporal programs. This study establishes an intracellular mechanism for posttranscriptional regulation of progressive fate determination in cortical neurogenesis.

## Graphical abstract

Syncrip orchestrates late‐stage neuronal lineage decisions in the developing neocortex by stabilizing critical transcripts via phase separation. Loss of Syncrip impedes superficial neuron formation, reshaping temporal fate trajectories and disrupting cortical lamination. These alterations result in cognitive deficits and highlight Syncrip's posttranscriptional control as a potential therapeutic target for neurodevelopmental disorders.

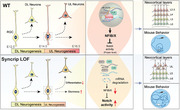

## 1 Introduction

The neocortex is a critical mammalian brain structure responsible for increased cognitive functions such as language, memory, and spatial navigation. The development of the neocortex is regulated by precise temporal developmental programs.^[^ ^1^, ^2^, ^3^ ^]^ In mammals, neural progenitor cells (NPCs) include RGCs, also known as apical progenitors (APs), which are predominantly found in the ventricular zone (VZ), and different populations of intermediate progenitors (IPs), also known as basal progenitors (BPs), which are predominantly found in the subventricular zone (SVZ), have a more limited self‐renewal capacity and developmental potential. RGCs give rise to six layers of neurons at different stages of neocortical development in an inside‐out manner, a process known as temporal patterning. This process relies on various transcriptional programs and posttranscriptional regulatory mechanisms to ensure the proper development of neurons.^[^ ^4^, ^5^, ^6^ ^]^ However, how RGCs shift from producing deep‐layer neurons to producing superficial neurons remains poorly understood. Abnormal neurodevelopment is linked to neurodevelopment disorders (NDDs), including autism spectrum disorder (ASD) and intellectual disability (ID).^[^ ^7^ ^]^ Investigating neocortical developmental processes and the associated molecular mechanisms will elucidate the pathogenesis of NDDs and aid in identifying potential therapeutic targets.

During cortical development, RGCs must rapidly alter their transcriptional programs through a sequential temporal process to ensure the continuous generation of cortical neurons. Single‐cell RNA sequencing (scRNA‐seq) has provided insights into the temporal transcriptional states of mammalian cortical RGCs,^[^ ^6^ ^]^ but further research is needed to understand how this continuous developmental pattern is established. Studies of conserved genes across species suggest that *Drosophila* neurons also undergo temporal developmental regulation. Furthermore, neuronal development in Drosophila has established an effective binary model. Tzumin Lee reported that Syncrip, along with Imp, is essential for neural temporal development in *Drosophila*, guiding neuroblast fate through a temporal gradient and influencing the formation of neuronal lineages.^[^ ^8^ ^]^

Syncrip, an RNA binding protein (RBP) also known as heterogeneous nuclear ribonucleoprotein Q (hnRNP Q),^[^ ^9^ ^]^ determines the fate of late‐born neurons by downregulating Chinmo in *Drosophila*.^[^ ^8^ ^]^ Furthermore, Syncrip is involved in various developmental processes in *Drosophila*, such as neuronal temporal development,^[^ ^8^ ^]^ neuromuscular junction (NMJ) synaptic transmission,^[^ ^10^ ^]^ and morphological regulation.^[^ ^11^ ^]^ However, Syncrip has been relatively understudied in mammalian models, particularly regarding its role in neocortical neurogenesis. Mutations in Syncrip have frequently been identified in patients with NDDs. In 2012, Strom reported novel mutations in several genes highly associated with ID, including Syncrip.^[^ ^12^ ^]^ Numerous studies have reported that de novo mutations in Syncrip cause ID and ASD.^[^ ^7^, ^13^, ^14^, ^15^, ^16^, ^17^, ^18^ ^]^ These mutations often result in abnormal brain morphology, as evidenced by Magnetic Resonance Imaging (MRI) findings showing periventricular nodular heterotopia and widened subarachnoid spaces.^[^ ^7^ ^]^ These structural abnormalities underscore the critical role of Syncrip in cortical development. Despite extensive documentation of the association between Syncrip mutations and NDDs, the molecular mechanisms of Syncrip in mammalian cortical development remain unclear. A deeper understanding of the regulatory role of Syncrip in neurogenesis could provide critical insights into cortical morphogenesis and the pathogenesis of NDDs.

In this study, we developed a mouse model with an RGC‐specific knockout of Syncrip to replicate neurodevelopmental disorder phenotypes, including abnormal cortical structures and behavioral anomalies. Using multiomics sequencing, we revealed that Syncrip acts as a core modulator in regulating RGCs within neocortical developmental programs that influence temporal fate decisions. We further propose that the Syncrip‐mediated post‐transcriptional regulatory signaling pathway is crucial for ensuring the production of late‐stage neurons by RGCs. Additionally, we explored the relationship between pathogenic Syncrip mutations and phase separation, providing new insights into the mechanisms of phase separation in development‐related diseases. In summary, this provides a novel perspective on mammalian neurogenesis, particularly in cortical development, and lays the foundation for future research into neurodevelopmental processes.

## 2 Results

### 2.1 Deletion of Syncrip causes Abnormal Neocortical Development

We compared the genomic and amino acid sequences of Syncrip and found it to be highly conserved among *Homo sapiens*, *Mus musculus*, and *Drosophila melanogaster*, featuring an hnRNP Q acidic domain (AcD) and three RNA recognition motifs (RRMs) (Figure S1A–C, Supporting Information). To investigate the expression of Syncrip in NPCs during mouse embryonic development, we analyzed a published single‐cell RNA sequencing (scRNA‐seq) database.^[^ ^19^ ^]^ Syncrip is widely expressed in APs, BPs, early neurons (ENs) and late neurons (LNs) (Figure S2A, Supporting Information). Additionally, we performed immunofluorescence staining of the dorsal neocortex at embryonic day (E) 12.5, E14.5, and E16.5, co‐labeling the samples with various cell markers (Figure S2B,C, Supporting Information). Syncrip is consistently expressed at high levels throughout mammalian neurodevelopment. To further investigate the function of Syncrip in neural development, we used Emx1‐Cre to specifically knockout Syncrip in dorsal neocortex NPCs. We inserted loxP sequences flanking exons 2, 3, and 4 of Syncrip and crossed them with Emx1‐Cre mice to obtain Syncrip^fl/fl^; Emx1^Cre^ mice (Figure S2D, hereafter referred to Syncrip conditional knockout mice [cKO]). We validated the knockout at both RNA and protein levels (Figure S2E–G, Supporting Information).

**Figure 1:**
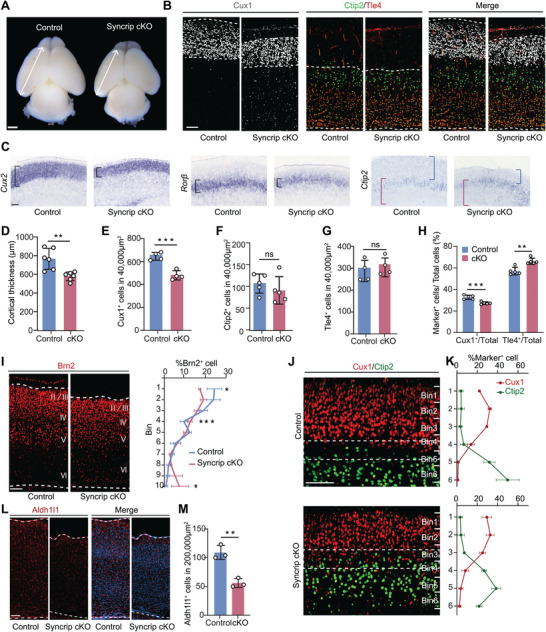
Deletion of Syncrip Causes Abnormal Neocortical Development. A) Brain dissection revealed that deletion of Syncrip caused defects in the dorsal forebrain at P3. B) Immunofluorescence staining of the Cux1, Ctip2 and Tle4 in coronal sections of the control (left) and Syncrip cKO (right) P3 neocortex. C) In situ hybridization (ISH) of *Cux2*, *Rorβ* and *Ctip2* in P7 control and Syncrip cKO coronal brain sections. D) Quantification of cortical thickness (control: n = 6, Syncrip cKO: n = 6). E–G) Quantification of Cux1^+^ cells (control: n = 4, Syncrip cKO: n = 4), Ctip2^+^ cells (control: n = 5, Syncrip cKO: n = 5), Tle4^+^ cells (control: n = 4, Syncrip cKO: n = 4) in the neocortex. H) Quantification of Cux1^+^ cells/total cells and Tle4^+^ cells/total cells (control: n = 4, Syncrip cKO: n = 4). I) Immunofluorescence staining of the Brn2 in coronal sections of control (left) and Syncrip cKO (right) P3 neocortices. P3 brains were quantified for the distribution of Brn2^+^ cells by subdividing the neocortex into 10 bins (control: n = 3, Syncrip cKO: n = 3). J,K) Immunofluorescence staining of Cux1 and Ctip2 in coronal sections of the control (top) and Syncrip cKO (bottom) P3 neocortex. P3 brains were quantified for the distribution of Cux1^+^ cells and Ctip2^+^ cells by subdividing part of the neocortex into 6 bins (control: n = 3, Syncrip cKO: n = 3). L) Immunofluorescence staining of Aldh1l1 in coronal sections of the control (left) and Syncrip cKO (right) P7 neocortex. M) Quantification of Aldh1l1^+^ cells in the neocortex (control: n = 3, Syncrip cKO: n = 3). Scale bars, 2 mm (A), 100 µm (B, C, I, J, L). The data are presented as the mean ± SEM. Statistical significance was determined via paired two‐tailed Student's *t* tests in (D) and unpaired two‐tailed Student's *t* tests in the other experiments. ^*^ *p* < 0.05, ^**^ *p* < 0.01, ^***^ *p* < 0.001.

We dissected and observed the postnatal day (P) 3 brains of Syncrip cKO mice and found that the telencephalon of Syncrip cKO mice was smaller than that of control littermates (**Figure** **1**A), with a 25% reduction in cortical thickness (578.8 µm ± 18.77 in the cKO mice versus 766.3 µm ± 45.76 in the control mice) (Figure 1B,D), particularly in the superficial layers (Figure 1B). Immunofluorescence revealed a 30% reduction in Cux1^+^ neuron density (a layer II‐IV marker) (484 ± 21.12 in cKO mice versus 658 ± 17.8 in control mice) (Figure 1E). In contrast, the densities of Ctip2^+^ (a layer V marker), Sox5^+^ (a layer V‐VI marker), Tle4^+^, and Tbr1^+^ (a layer VI marker) neurons remained unchanged (Figure 1B,F,G; Figure S2H, Supporting Information). In situ hybridization revealed decreased densities of *Cux2^+^* (a layer II‐IV marker) and *Rorβ^+^* (a layer IV marker) cells (Figure 1C). By calculating the percentages of superficial (layers II‐IV) and deep (layers V‐VI) neurons within the entire cortical distribution, we found that the percentage of superficial layer neurons (Cux1^+^ cell count/total cell count) decreased from 32.80% ± 0.84% to 27.34% ± 0.53%, while the percentage of deep layer neurons (Tle4^+^ cell count/total cell count) increased from 57.10% ± 1.59% to 66.17% ± 1.28% (Figure 1H).

When we stained for Brn2, a marker specifically distributed in the neocortex at layers II‐III and V,^[^ ^20^, ^21^ ^]^ we observed that Brn2^+^ cells were sparser at Bin4 in the control. However, in the Syncrip cKO mouse neocortex, Brn2^+^ cells at Bin4 were not sparser but instead exhibited a more uniform distribution (Figure 1I). To investigate whether the abnormal distribution of Brn2^+^ cells was associated with defective cortical layer formation, we performed costaining for Cux1 and Ctip2. We found that the interval between Cux1^+^ and Ctip2^+^ layers was absent in Syncrip cKO mice (Figure 1J,K). In summary, the loss of Syncrip leads to abnormal cortical development, specifically associated with a reduction in superficial layer neurons.

Given that astrocyte generation occurs after neurogenesis,^[^ ^22^, ^23^ ^]^ we next examined whether Syncrip deficiency affects astrocyte development. We quantified astrocytes in the neocortex of Syncrip cKO mice using the astrocyte marker Aldh1l1^[^ ^24^ ^]^ and found that their density was reduced by half compared to controls (56 ± 4.58 in cKO mice versus 109 ± 7.10 in control mice) (Figure 1L,M).

To explore the widespread effects of Syncrip loss, we used Nestin‐Cre, which is broadly expressed in the nervous system, to specifically knock out Syncrip. Syncrip^fl/fl^; Nestin^Cre^ mice exhibited abnormal development, with significantly reduced body weights and sizes (Figure S2I–K, Supporting Information). Knockout efficiency was confirmed by staining with a Syncrip antibody (Figure S2L, Supporting Information), the complete loss of Syncrip signal in Nestin‐Cre‐mediated cKO mice further confirms the antibody's specificity for immunofluorescence applications. Moreover, abnormal neocortex lamination was observed, characterized by a reduction in the superficial layers, mirroring the phenotype seen in Emx1‐Cre mediated Syncrip cKO mice (Figure S2M). These data confirm the critical role of Syncrip in the development of the superficial layers of the neocortex.

### 2.2 Syncrip Affects the Fate Determination of Late‐Stage Neurogenesis

**Figure 2:**
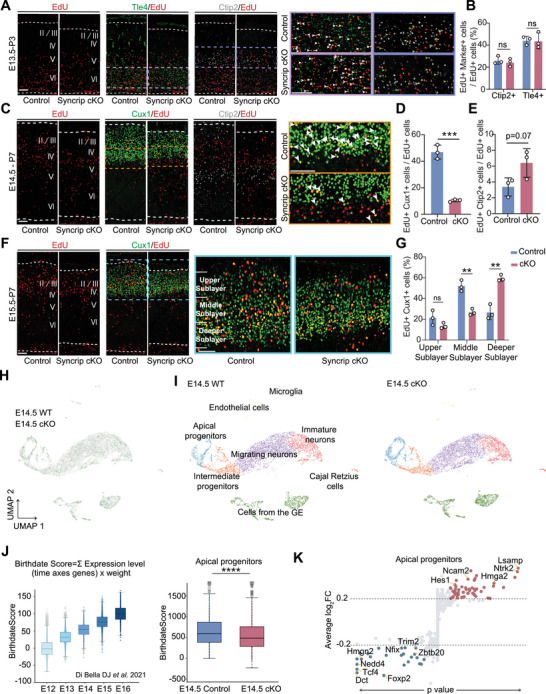
Syncrip Determines Late NPCs Fate in the Developing Neocortex. A) Immunofluorescence staining for EdU, Ctip2 and Tle4 in coronal sections of the control and Syncrip cKO P3 neocortices. EdU was administered at E13.5. The white arrowheads indicate EdU^+^ Tle4^+^ cells, and the green arrowheads indicate EdU^+^ Ctip2^+^ cells. B) The ratio of EdU^+^ Marker^+^ cells to EdU^+^ cells in the neocortex was quantified (control: n = 3, Syncrip cKO: n = 3). C) Immunofluorescence staining for EdU, Cux1 and Ctip2 in coronal sections of the control and Syncrip cKO P7 neocortices. EdU was administered at E14.5. The white arrowheads indicate EdU^+^ Cux1^+^ cells. D,E) Quantification of the ratio of EdU^+^ Cux1^+^ cells to EdU^+^ cells, EdU^+^ Ctip2^+^ cells to EdU^+^ cells (control: n = 3, Syncrip cKO: n = 3). F) Immunofluorescence staining for EdU and Cux1 in coronal sections of control and Syncrip cKO P7 neocortices. EdU was administered at E15.5. G) In (F), the region containing Cux1^+^ cells were divided into three sublayers. The ratios of Cux1^+^ EdU^+^ cells to total EdU^+^ cells in each sublayer were quantified (control: n = 3, Syncrip: cKO n = 3). H) UMAP visualization of a scRNA‐seq dataset containing neocortical cells from E14.5 control and Syncrip cKO mice. I) UMAP plot of neocortical cells from both control and Syncrip cKO mice. J) Wu's model was used to evaluate cell birthdate scores, and Arlotta's scRNA‐seq was used to quantify birthdate scores for APs in the control and Syncrip cKO E14.5 neocortices. K) A symmetric volcano plot illustrates differentially expressed genes in apical progenitors between E14.5 cKO and E14.5 WT scRNA‐seq. The x‐axis represents signed log_10_‐adjusted *p*‐values (genes with positive fold changes appear on the right, while those with negative fold changes appear on the left), and the y‐axis shows average log_2_ fold changes. Genes meeting both criteria (*p*_val_adj < 0.05 and |log_2_ FC| > 0.2) are colored to indicate upregulation (red) or downregulation (blue‐green), whereas non‐significant genes are shown in gray. Selected genes are labeled for clarity, and dashed lines mark the log_2_ fold change thresholds (±0.2). This analysis excluded sex genes. Scale bar, 100 µm. The data are presented as the means ± SEMs. Statistical significance was determined via an unpaired two‐tailed Student's *t* test. ^*^ *p* < 0.05, ^**^ *p* < 0.01, ^***^ *p* < 0.001, ^****^ *p* < 0.0001.

Since neocortical layers are generated from RGCs in a temporal order, we conducted birthdating analysis with extended 5‐ethynyl‐2′‐deoxyuridine (EdU) labeling to further investigate RGC fate during neurogenesis. We injected EdU at E13.5 and co‐labeled P3 brain tissue sections with Tle4 and Ctip2. No significant difference was observed between Syncrip cKO and control mice (**Figure** **2**A,B). We injected EdU at E14.5 followed by colabeling with Cux1 and Ctip2 at P7. In the neocortex of Syncrip cKO mice, there was a 79% decrease in the number of E14.5‐labeled EdU‐labeled cells costained with Cux1 (10.38% ± 0.56% in the cKO mice versus 46.93% ± 3.04% in the control mice) (Figure 2C,D) and a trend toward an increase in the number of EdU‐labeled cells costained with Ctip2 (6.40% ± 1.04% in the cKO mice versus 3.38% ± 0.66% in the control mice) (Figure 2C,E), suggesting that superficial neuronal fate production is impeded by Syncrip deletion. Similarly, when EdU was injected at E15.5 and costained with Cux1 at P7, the proportion of EdU^+^ cells in the middle sublayer of the superficial layers were reduced by half compared to controls (26.82% ± 1.76% in cKO mice versus 52.01% ± 3.09% in control mice), while the proportion in the deeper sublayer doubled (59.60% ± 1.75% in cKO mice versus 26.64% ± 4.10% in control mice) (Figure 2F,G).

A temporal transcriptional program in neocortical progenitors enables specification of neuronal types.^[^ ^25^ ^]^ To confirm the altered temporal fate of RGCs, we collected neocortical tissue from Syncrip cKO mice at E14.5 for scRNA‐seq analysis. After filtering out low‐quality cells, unsupervised clustering analysis based on molecular marker expression revealed eight major cell clusters: apical progenitors, intermediate progenitors, migrating neurons, immature neurons, interneurons (cells from the ganglionic eminence [GE]), microglia, Cajal–Retzius cells, and endothelial cells (Figure 2H,I; Figure S3, Supporting Information). Wu et al. predicted the date of birth of each cell by performing an ordered regression model of temporal gene expression levels, a process known as calculating the birthdate score.^[^ ^26^ ^]^ We applied this method to calculate the birthdate scores of APs from E12–E16 using scRNA‐seq data published by Arlotta.^[^ ^5^ ^]^ We found that this method accurately reflected the temporal dynamics of APs, indicating that cells that produce earlier‐born neurons receive lower birthdate scores and generate later‐born neurons that receive higher scores. Next, we calculated the birthdate scores of APs from Syncrip cKO mice and found that these cells received lower scores compared to control mice, indicating that Syncrip knockout causes APs to exhibit earlier transcriptional characteristics and altered temporal developmental states (Figure 2J).

We then used a maturation level calculation method utilizing a time series dataset from Telley.et al^[^ ^6^ ^]^ and trained a regression model to predict maturation scores for control and Syncrip cKO in our dataset.^[^ ^27^ ^]^ APs in Syncrip cKO mice exhibited lower maturation scores compared to those in control mice (Figure S4A, Supporting Information). These results suggest that Syncrip deletion alters the temporal fate of APs, leading to abnormalities in their late‐stage fate, possibly due to the disruption of temporal transcriptional programs.

To further explore altered temporal genes, we analyzed differential expressed genes (DEGs) via scRNA‐seq of APs and identified several genes with elevated expression at early stages of neurogenesis, such as Hmga2 and Hes1,^[^ ^6^ ^]^ as well as genes with reduced expression at later stages, such as Nedd4 and Zbtb20^[^ ^6^ ^]^ (Figure 2K; Figure S4B, Supporting Information). To confirm these temporal genes alterations in NPCs, we performed RNA‐seq on NPCs derived from Syncrip cKO mice and found that the temporal gene alterations were consistent with those identified via scRNA‐seq, with the expression of early‐stage genes, such as Top2a, Hes1, and Hmga2,^[^ ^6^ ^]^ increasing after Syncrip knockout, whereas the expression of late‐stage genes, such as Dbi, Zbtb20, and Nedd4,^[^ ^6^ ^]^ decreased (Figure S4C–H, Supporting Information).

### 2.3 Syncrip Deficiency Maintains the Early Fate of RGCs in the Developing Neocortex

**Figure 3:**
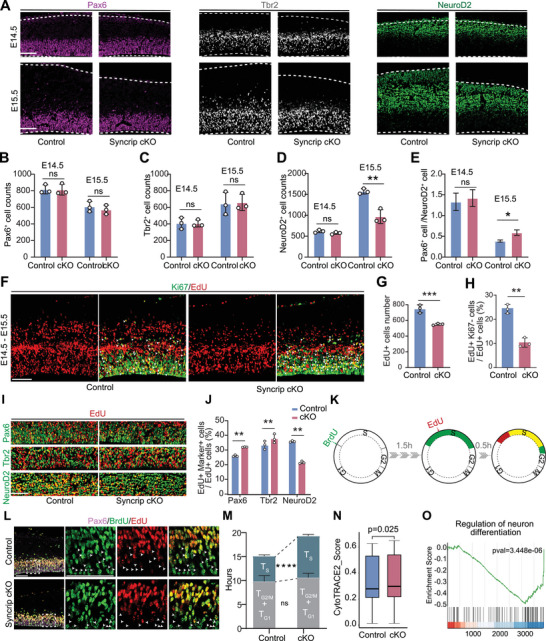
Syncrip Defects Affect Neural Progenitor Cell Dynamics and Cell Cycle Progression in Developing Neocortices. A) Pax6, Tbr2 and NeuroD2 staining of coronal sections from control and Syncrip cKO E14.5 and E15.5 neocortical regions. B–D) Quantification of Pax6^+^, Tbr2^+^ and NeuroD2^+^ cell counts in the neocortex (control: n = 3, Syncrip cKO: n = 3). E) Quantification of the proportion of Pax6^+^/NeuroD2^+^ cells in the neocortex (control: n = 3, Syncrip cKO: n = 3). F) Cell cycle exit analysis. Double labeling of the mitotic marker Ki67 and EdU in E15.5 coronal sections. The white arrowheads indicate EdU^+^ Ki67^+^ cells. G) Quantification of EdU^+^ cells in the neocortex (control: n = 3, Syncrip cKO: n = 3). H) Quantification of the proportion of Ki67^−^ EdU^+^/EdU^+^ cells in the neocortex (control: n = 3, Syncrip cKO: n = 3). I) Double staining of EdU and Pax6, Tbr2, NeuroD2 in E15.5 coronal sections. J) Quantification of the proportion of marker^+^ EdU^+^/EdU^+^ cells in coronal sections of the control and Syncrip cKO E15.5 neocortices (control: n = 4, Syncrip cKO: n = 4). K) Scheme of the experimental analysis of the cell cycle. L) BrdU, EdU and Pax6 staining of E14.5 coronal sections of control and Syncrip cKO E14.5 neocortices. The white arrowheads indicate BrdU^+^ EdU^−^ cells. M) Quantification of NPCs in different phases of the cell cycle in the neocortex of control and Syncrip cKO mice. N) Cell stemness analysis via the CytoTRACE 2 method. O) GSEA revealed that the expression of “regulation of neuron differentiation” genes decreased in Syncrip cKO NPCs. Scale bar, 100 µm. The data are presented as the mean ± SEM. Statistical significance was determined via unpaired two‐tailed Student's *t* test (B, C, D, E, G, H, M), via paired two‐tailed Student's *t* test (J), or via the Wilcoxon rank‐sum test for Cytotrace2. ^*^ *p* < 0.05, ^**^ *p* < 0.01, ^***^ *p* < 0.001, ^****^ *p* < 0.0001.

To further investigate whether the reduction in superficial layer neurons in Syncrip cKO mice was due to a decrease in RGC numbers, we evaluated the expression of RGCs marker Pax6 and IPs marker Tbr2 in Syncrip cKO and littermate control neocortices at E14.5 and E15.5. At E14.5, no significant differences in stem cell numbers were observed between the cKO and control groups (**Figure** **3**A–E). However, by E15.5, the proportions of NPCs and neurons significantly changed (Figure 3A–E). To test whether apoptosis is increased in the neocortex of Syncrip cKO mice, we performed TdT‐mediated dUTP Nick‐End Labeling (TUNEL) staining and found that there was no significant cell death in the neocortex during these stages (Figure S5A, Supporting Information). To detect changes in NPCs after E14.5, we intraperitoneally injected EdU into pregnant mice at E14.5 and collected embryonic brains at E15.5. EdU^+^ cells were reduced by 30% in Syncrip cKO mice (Figure 3F,G). Colabeling with the mitotic marker Ki67 revealed a reduction in the proportion of NPCs exiting the cell cycle from 24.60% ± 1.27% to 10.44% ± 1.34% (Figure 3F,H). We then costained EdU with NPCs and neuron markers. Syncrip deficiency resulted in an increase in the percentage of RGCs (Pax6^+^ EdU^+^/EdU^+^) and IPs (Tbr2^+^ EdU^+^/EdU^+^) from 26.01% ± 0.36% to 32.06% ± 0.07% and 33.39% ± 1.64% to 37.82% ± 1.76%, respectively, with a decrease in the percentage of neurons (NeuroD2^+^ EdU^+^/EdU^+^) from 35.85% ± 0.29% to 21.57% ± 0.49% (Figure 3I,J). To verify whether the transition of Syncrip cKO RGCs to IPs was altered, we costained for Pax6 and Tbr2 at E15.5. The percentage of Pax6^+^Tbr2^+^ cells in Syncrip cKO mice was unchanged (Figure S5B, Supporting Information). The absence of Syncrip appears to shift RGC fate, leading to reduced differentiation and the retention of more cells in a stem‐like state.

Previous studies have indicated that the duration of the cell cycle reflects the fate of RGCs.^[^ ^28^ ^]^ To investigate whether Syncrip deletion affects the cell cycle in RGCs, we stained E14.5 Syncrip cKO brain tissue sections with the M phase marker phospho‐histone H3 (PH3) and observed no significant differences (Figure S5C, Supporting Information). We then used EdU and 5‐bromo‐2′‐deoxyuridine (BrdU) double labeling for detailed analysis (Figure 3K). Syncrip cKO RGCs exhibited a prolonged duration of the overall cell cycle (control: Tc = 17.12 h ± 0.40; Syncrip cKO: Tc = 21.30 h ± 0.34) and S phase (control: Ts = 5.33 h ± 0.09; Syncrip cKO: Ts = 8.70 h ± 0.12) (Figure 3L,M). We also performed cell cycle scoring using Seurat and analyzed the AP clusters (Figure S5D, Supporting Information). Syncrip cKO APs had a higher proportion of cells in the S phase, further indicating that Syncrip deletion prolongs the S phase in APs.

To determine whether Syncrip deletion affects stemness, we used developmental potential prediction (CytoTRACE 2) to analyze NPCs.^[^ ^29^ ^]^ Consistent with our cell cycle findings, Syncrip cKO NPCs exhibited enhanced stemness (Figure 3N). Next, we performed RNA‐seq on Syncrip cKO and control E14.5 NPCs for Gene Ontology (GO) and gene set enrichment analysis (GSEA). In Syncrip cKO NPCs, we observed an upregulation of genes related to cell division, including spindle checkpoint signaling, chromosome segregation, and the meiotic cell cycle (Figure S5E,G, Supporting Information), while genes related to neuron differentiation, axon development, and neuron development were downregulated (Figure 3O; Figure S5F, Supporting Information).

### 2.4 Syncrip Binds the 3′UTRs of *Nfib* and *Nfix* to Regulate their Stability

**Figure 4:**
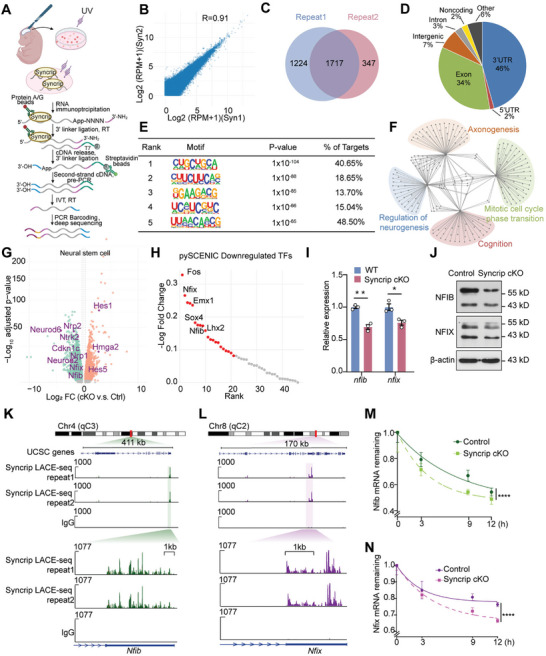
Syncrip Binding to the 3′ UTRs of *nfib* and *nfix* Modulates mRNA Stability in E14.5 NPCs. A) Experimental analysis scheme of LACE‐seq, adapted from Su R, et al. B) LACE‐seq replicate correlations for usable reads per gene normalized to coverage (reads per million reads mapped, RPM) for Syncrip in NPCs. The coefficient R value was given by the two‐tailed Pearson's correlation. C) Venn diagram of two biological replicates of Syncrip‐binding peaks. D) Genomic distribution of the Syncrip LACE‐seq peaks in E14.5 NPCs. E) Syncrip‐binding motifs identified via LACE‐seq using HOMER. F) Network analysis of the enriched GO terms of Syncrip‐specific targets. G) Volcano plot of genes upregulated (orange) and downregulated (green) in E14.5 Syncrip cKO NPCs compared with control, as analyzed using DESeq2. H) PySCENIC was used to assess the activity of transcription factors in APs and rank transcription factors with reduced activity. I) qPCR analysis of *nfib* and *nfix* mRNA expression in NPCs from control and Syncrip cKO E14.5 neocortices (control: n = 3, Syncrip cKO: n = 3). J) Immunoblots of NFIB, NFIX and β‐actin in extracts of NPCs from control and Syncrip cKO E14.5 neocortices. K,L) The Integrative Genomics Viewer was used to observe Syncrip LACE‐seq reads on chromosome 4 (Chr4) and chromosome 8 (Chr8). An enlarged view of the Syncrip‐binding sites at the *nfib* and *nfix* transcript is displayed at the bottom. M,N) qPCR analysis of nfib and nfix expression in E14.5 NPCs from control and Syncrip cKO mice at 0, 3, 9, and 12 h post actinomycin D treatment (control: n = 3, Syncrip cKO: n = 3). The data are presented as the mean ± SEM. Statistical significance was determined via an unpaired two‐tailed Student's *t* test. *p < 0.05, **p < 0.01, ***p < 0.001, ****p < 0.0001.

Previous studies have indicated that the biological function of the RBP Syncrip may be related to RNA processing.^[^ ^30^, ^31^ ^]^ To further explore how Syncrip impacts late transcriptional programs, we utilized linear amplification of complementary DNA ends and sequencing (LACE‐seq), a method capable of detecting RBP targets with low input cell numbers^[^ ^32^ ^]^ (**Figure** **4**A). We performed two replicates of LACE‐seq in isolated E14.5 cultured NPCs and found consistent results (Figure 4B), taking the intersection of the two replicates (Figure 4C). The majority of peaks appeared in the 3ʹUTR (46%), with the remainder located in 5ʹUTRs, introns, and coding exons (Figure 4D). This distribution, similar to previous individual‐nucleotide resolution crossLinking and immunoprecipitation (iCLIP) studies in rat neurons cultured in vitro,^[^ ^31^ ^]^ indicates that Syncrip functions primarily in a manner dependent on the 3′UTR. Among the enriched peaks, CUGCUGCA ranked highest among the motifs with a *p* value of 1 × 10^−104^, and 40.65% of the Syncrip peaks contain the CUGCUGCA motif; CUUCUUCAG ranks second, with a *p* value of 1 × 10^−88^, and 18.65% of the peaks contain this motif (Figure 4E). GO analysis revealed that Syncrip targets are involved in mitotic cell cycle phase transition, axonogenesis, regulation of neurogenesis, and cognition (Figure 4F).

To explore the downstream molecules regulated by Syncrip, we analyzed the scRNA‐seq data and bulk RNA‐seq data of E14.5 Syncrip cKO NPCs cultured in vitro. Consistent with the scRNA‐seq observations, there was an increase in genes associated with stemness maintenance and early RGC fate, such as Hes1, Hes5, and Hmga2, and a decrease in genes associated with neuronal differentiation and late neuronal fate, such as Neurod2, Nfix, Nfib and Zbtb20 (Figure 4G). Furthermore, alterations in transcription factors are crucial in determining the transcriptional program and fate of RGCs. To assess the impact on transcription factors during late‐stage cortical neurogenesis, we used pySCENIC to analyze transcription factor activity in APs via scRNA‐seq of control and cKO samples. By ranking transcription factors with reduced activity, we identified two transcription factors, Nfix and Nfib, which are associated with late‐stage neurogenesis^[^ ^33^, ^34^, ^35^ ^]^ (Figure 4H). In NPCs cultured from E14.5 Syncrip cKO mice, both the mRNA and protein levels of Nfib and Nfix were decreased (Figure 4I,J).

To investigate how Syncrip regulates *Nfib* and *Nfix* transcripts, we analyzed whether Syncrip binds to their mRNAs and found that Syncrip peaks were enriched at the 3′UTRs of both *Nfix* and *Nfib* mRNAs (Figure 4K,L). To verify whether Syncrip modulates the mRNA stability of *Nfib* and *Nfix*, we treated NPCs from control and Syncrip cKO mice with actinomycin D to inhibit transcription. After treatment for a certain period, *Nfib* and *Nfix* had shortened mRNA half‐lives and decreased mRNA stability in the absence of Syncrip (Figure 4M). During cortical development, RNA transcript stability is crucial for normal neural development, and its instability leads to anomalies in neurogenesis and aberrant temporal fate determination in NPCs.^[^ ^36^ ^]^

### 2.5 Nfib and Nfix Inhibit Notch Activity and are Crucial for the Establishment of Late Fate Decisions

**Figure 5:**
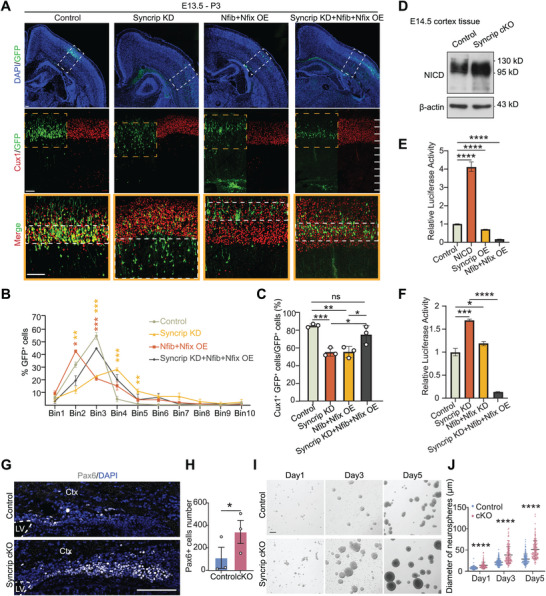
Overexpression of Nfib and Nfix Rescues Syncrip Knockdown Effects and Regulates the Notch Pathway. A) Control, Syncrip knockdown, and Nfib/Nfix overexpression plasmids were electroporated into the embryonic brain at E13.5. Brain sections were collected at P3 and stained for Cux1 (red) to identify upper‐layer neurons. In the displayed images, GFP (green) indicates electroporated cells. The middle row shows the individual GFP and Cux1 channels separately, while the bottom row presents the merged GFP and Cux1 signals. B) P3 brains were quantified for the distribution of GFP^+^ cells by subdividing the neocortex into 10 bins (n = 3). C) Quantification of the proportion of Cux1^+^ GFP^+^/GFP^+^ cells in the neocortex in coronal sections (n = 3). D) Immunoblots of NICD and β‐actin using extracts of control and Syncrip cKO E14.5 neocortices. E) Measurements of CBFRE luciferase activity after transfection with control, NICD, Syncrip overexpression or Nfib/Nfix overexpression vectors. n = 3 biological replicates. F) Measurements of CBFRE luciferase activity after transfection with control, Syncrip knockdown, Nfib/Nfix knockdown or Syncrip knockdown combined with Nfib/Nfix overexpression. n = 3 biological replicates. G) Immunofluorescence staining of the RGC marker Pax6 in coronal sections of the control (top) and Syncrip cKO (bottom) P3 neocortex. H) Quantification of Pax6^+^ cells in the neocortex (n = 3). I) Representative images at days 1, 3, and 5 of NPC neurosphere culture (third passage) derived from the control (top) and Syncrip cKO (bottom) E14.5 neocortex. Scale bar, 100 µm. J) Quantification of the diameter of neurospheres derived from the control and Syncrip cKO E14.5 neocortex at days 1, 3, and 5. Scale bar, 100 µm. The data are presented as the mean ± SEM. Statistical significance was determined via an unpaired two‐tailed Student's *t* test. ^*^ *p* < 0.05, ^**^ *p* < 0.01, ^***^ *p* < 0.001, ^****^ *p* < 0.0001.

To confirm whether the late fate commitment of RGCs is regulated by these two key transcription factors, we performed in utero electroporation (IUE) at E13.5 to introduce Nfix and Nfib overexpression plasmids or green fluorescent protein (GFP)‐expressing empty vectors into periventricular RGCs, and collected brain tissues at P3 (**Figure** **5**A). In the neocortex of P3 controls, GFP^+^ neurons were primarily located in layer IV. In contrast, Nfib and Nfix overexpression shifted GFP^+^ cells to layers II/III, indicating that RGCs produce later‐born neurons upon Nfib and Nfix overexpression (Figure 5A). In contrast, Syncrip knockdown (KD) via shRNA in the E13.5 neocortex resulted in RGCs producing fewer neurons, with their distribution more downward compared to the control, indicating a fate more similar to earlier‐stage RGCs (Figure 5A). Moreover, when Syncrip KD was cointroduced with Nfib and Nfix overexpression (OE) plasmids into the lateral ventricles at E13.5, GFP^+^ cells were primarily relocated to cortical layer IV, similar to the control (Figure 5A,B). We determined the percentage of GFP^+^Cux1^+^ cells, and both Syncrip KD (55.27% ± 2.70%) and Nfib and Nfix overexpression (55.52% ± 3.59%) resulted in a 30% reduction compared with the control (85.29% ± 1.08%) (Figure 5C). When Syncrip KD and Nfib and Nfix overexpression were simultaneously introduced, the proportion of GFP^+^ Cux1^+^ cells (74.92% ± 5.91%) was nearly restored to the control level (Figure 5C).

Nfib and Nfix have been reported to suppress Notch signaling in RGCs, and Notch signaling is activated in mice lacking both genes.^[^ ^37^, ^38^, ^39^ ^]^ Considering the observation in Syncrip cKO mice of increased NPCs and decreased neurons at E14.5‐E15.5, and EdU‐labeled cells, along with RNA‐seq data, showing elevated Hes1 and Hes5 expression in Syncrip cKO NPCs (Figure 4G), these data indicate that Syncrip deficiency might activate the Notch signaling pathway, suggesting a direct link between Syncrip‐Nfib‐Nfix and Notch signaling. To verify this hypothesis, we extracted proteins from the neocortices of control and Syncrip cKO mice and assessed Notch Intracellular Domain (NICD) expression via Western blot. Syncrip cKO mice showed increased NICD expression compared to control mice (Figure 5D). We performed dual‐luciferase reporter assays with CBF1‐responsive element (CBFRE) as the reporter gene for detecting Notch signaling pathway activity. Overexpression of Syncrip along with Nfib and Nfix significantly decreased luciferase activity (Figure 5E), whereas knockdown of Syncrip, along with Nfib and Nfix, increased luciferase activity (Figure 5F). Notably, overexpression of Nfib and Nfix reversed the activation of Notch signaling caused by Syncrip knockdown (Figure 5F). These findings further confirmed that Syncrip‐Nfib/Nfix‐Notch signaling acts as an axis to regulate neocortical neurogenesis.

To further observe the phenotype of Notch signaling activation caused by Syncrip deletion, we stained P3 Syncrip cKO neocortex sections with Pax6. More Pax6^+^ cells were located around the lateral ventricle (LV) (Figure 5G,H), indicating that some cells maintained stemness postnatally in Syncrip cKO mice. Additionally, to determine whether stemness was altered in E14.5 NPCs from Syncrip cKO mice, we isolated E14.5 NPCs from both control and Syncrip cKO mice and cultured them as neurospheres in vitro, by the third passage, more NPCs and larger neurospheres were observed in Syncrip cKO cultures (Figure 5I,J), suggesting enhanced stemness and proliferation.

Therefore, Syncrip facilitates this transition by rapidly inhibiting Notch activity through posttranscriptional regulation of Nfib and Nfix at E14.5, thereby contributing to the temporal progression of NPC fate.

### 2.6 Syncrip Deficiency Leads to Spatial Learning Memory Impairment and Decreased Desire to Explore

To explore whether Syncrip cKO mice replicate the abnormalities observed in patients with NDDs, we evaluated the behavioral and cognitive functions of Syncrip cKO mice using various tests, including the Morris water maze, Y maze, open field, elevated plus maze, and three‐chamber assays.

**Figure 6:**
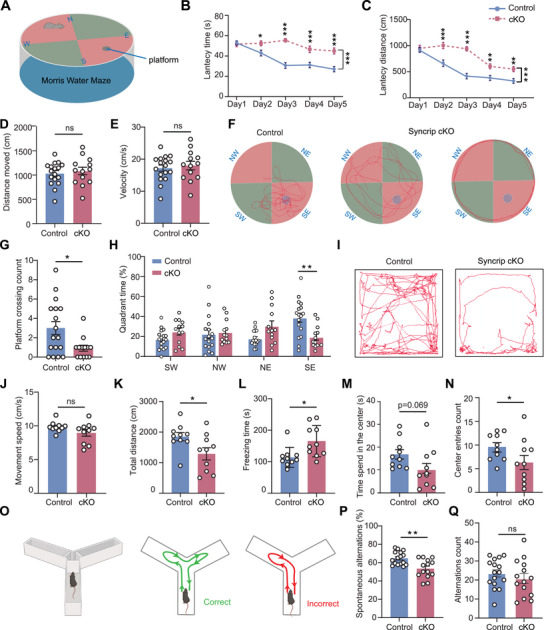
Impact of Syncrip Deletion on Spatial Learning, Memory, and Exploratory Behavior in Mice. A) Experimental scheme of the Morris water maze test. B,C) Latency time (B) and distance (C) to find the hidden platform across the training period in the Morris water maze test. Control: n = 17, Syncrip cKO: n = 13. (D,E) Bar charts of the distance moved (D) and velocity (E) during the probe trial. (F) Overhead view of the Morris water maze showing the swimming trajectories of control and Syncrip cKO mice during the probe trial (platform removed). The platform was in the southeast (SE) quadrant. (G) The frequency of platform crossings. (H) Time spent in each quadrant. Control: n=17, Syncrip cKO: n=13. I) Representative trajectories of control and Syncrip cKO mice in the open‐field arena. J–N) Quantification of the movement speed, total distance traveled, freezing time, the number of entries into the center, and the time spent in the center of control and Syncrip cKO mice in the open‐field arena. Control: n = 10, Syncrip cKO: n = 10. O) Experimental scheme of the Y maze. P,Q) Quantification of the total number of alternations and the ratio of spontaneous alternations in the Y maze. Control: n = 16, Syncrip cKO: n = 14. The data are presented as the means ± SEMs. Statistical significance was determined using two‐way ANOVA followed by Sidak's multiple comparisons test (B, C) or an unpaired two‐tailed Student's *t*‐test (D‐E, G‐H, J‐N, P‐Q). ^*^ *p* < 0.05, ^**^ *p* < 0.01, ^***^ *p* < 0.001.

In the Morris water maze (**Figure** **6**A), Syncrip cKO mice took significantly more time and traveled farther to find the platform over five consecutive days (Figure 6B,C), indicating impaired spatial learning. In the Day 6 probe test, although there were no significant differences in swimming speed or distance between the control and Syncrip cKO mice (Figure 6D,E), the Syncrip cKO mice crossed the original platform location fewer times (0.92 ± 0.31% in the cKO mice versus 3.00 ± 0.68% in the control mice) and had a reduced percentage of time spent in the target quadrant (20.49% ± 3.94% in the cKO mice versus 39.06% ± 4.51% in the control mice) (Figure 6F–H). In the open field test (Figure 6I), Syncrip cKO mice showed no significant changes in movement speed (Figure 6J). However, their total distance traveled significantly decreased (1287 cm ± 202.2 in the cKO mice versus 1852 cm ± 135.6 in the control mice) (Figure 6K), and their freezing time significantly increased (165.5 s ± 15.78 in the cKO mice versus 113.9 s ± 10.03 in the control mice) (Figure 6L). They also exhibited hesitation in exploring the center area, as shown by the decreased time spent in the center (Figure 6M,N). In the Y‐maze test (Figure 6O), Syncrip cKO mice presented lower spontaneous alternation percentages without a significant difference in alternation count (53.49% ± 2.72% in the cKO mice versus 64.68% ± 1.79% in the control mice) (Figure 6P,Q). Additionally, no significant differences were observed in the elevated plus maze test or the three‐chamber test (Figure S6, Supporting Information).

In summary, these behavioral tests revealed that Syncrip deletion in mice results in spatial memory impairments, reduced exploratory behavior and short‐term memory deficits, mirroring the ID observed in patients with Syncrip‐associated NDDs.

### 2.7 Pathogenic Syncrip Mutations Disrupt the Phase Separation and Protein‐Binding Characteristics of the Encoded Protein

Syncrip is detected in both the nucleus and cytoplasm (Figure S2B, Supporting Information), as demonstrated by our results and consistent with previous reports,^[^ ^31^ ^]^ and has been reported to localize to P‐bodies and form cytoplasmic condensates.^[^ ^40^, ^41^ ^]^ To investigate this further, we costained Syncrip and the P‐body marker DCP1A and found that Syncrip colocalized with DCP1A, suggesting that Syncrip may be present in P‐bodies, a type of membraneless organelle formed by phase separation that stabilizes internal RNA transcripts^[^ ^42^ ^]^ (Figure S7A, Supporting Information). We also performed in vitro coimmunoprecipitation experiments and reported that Syncrip can recruit PABPC1 and HuR (Figure S7B, Supporting Information), proteins crucial for RNA stability and downstream RNA regulation that are also confirmed to reside in P‐bodies.

To verify whether Syncrip can undergo phase separation, we predicted the SYNCRIP protein sequence and identified C‐terminal intrinsically disordered regions (IDRs) (Figure S7C, Supporting Information), which are known to mediate liquid‒liquid phase separation.^[^ ^43^ ^]^ We then purified EGFP‐tagged SYNCRIP proteins from human and mouse origins and observed that these proteins formed condensates in vitro (Figure S7D,E, Supporting Information). Focusing on the human EGFP‐ SYNCRIP protein, we found that the resulting condensates exhibited substantial fluidity and displayed the characteristic droplet fusion events typical of liquid–liquid phase separation, as observed by microscopy (Figure S7F, Supporting Information).

**Figure 7:**
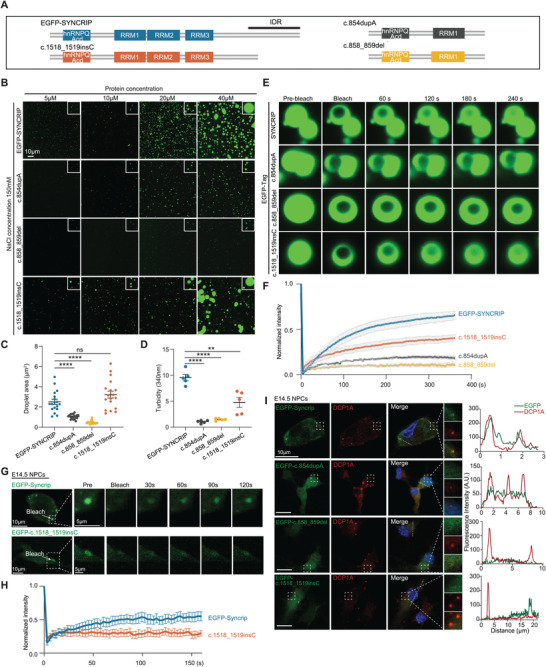
SYNCRIP Undergoes Phase Separation with a Reduced Capacity in Disease‐causing Mutants. A) Schematic representation of EGFP‐SYNCRIP and its pathogenic mutations. B) Phase plot of EGFP‐SYNCRIP and its disease‐causing mutants across various protein concentrations with a salt concentration of 150 mM. Scale bar, 10 µm. C) The average droplet area (NaCl concentration: 150 mM; protein concentration: 40 µM) was quantified. D) The turbidity was quantified at 340 nm (NaCl concentration: 150 mM; protein concentration: 40 µM). E,F) FRAP assay showing the fluorescence recovery of EGFP‐SYNCRIP and its disease‐causing mutants after photobleaching with a high‐intensity laser. Quantification of FRAP assays for EGFP‐SYNCRIP and its disease‐causing mutant droplets (n = 10). G) FRAP assay of E14.5 primary NPCs transfected with LV‐Syncrip and LV‐c.1518_1519insC. 10 µm for the overview images, 5 µm for the magnified regions. H) Quantification of FRAP assays for EGFP‐SYNCRIP and its disease‐causing mutant droplets (n = 5). I) Co‐localization analysis of Syncrip and its pathogenic mutations with DCP1A in E14.5 primary NPCs. E14.5 NPCs were transfected with LV‐Syncrip, LV‐c.854dupA, LV‐c.858_859del, or LV‐c.1518_1519insC and stained with DCP1A (red). Dashed boxes indicate regions of interest magnified in the inset. Fluorescence intensity profiles were quantified along the indicated lines in the insets and plotted for EGFP (green) and DCP1A (red). Scale bar, 10 µm. The data are presented as the mean ± SEM. Statistical significance was determined via an unpaired two‐tailed Student's *t* test. ^**^ *p* < 0.01, ^****^ *p* < 0.0001.

NDDs have been closely associated with phase separation in previous research.^[^ ^44^ ^]^ However, the underlying mechanisms remain elusive. In NDD patients with SYNCRIP mutations, such as c.858_859del^[^ ^7^ ^]^ and c.854dupA,^[^ ^7^ ^]^ the mutations result in protein truncation, leading to the loss of the RRM2, RRM3, and C‐terminal IDR regions (**Figure** **7**A; Figure S7G,H, Supporting Information). Patients exhibit ID and developmental disorders, with the c.858_859del mutation also displaying ASD symptoms and the c.854dupA mutation accompanied by epileptic seizures. Interestingly, another mutation, c.1518_1519insC,^[^ ^7^ ^]^ occurs within the C‐terminal IDR region, causing partial truncation of the C‐terminal IDR region but not affecting the functional domains of SYNCRIP (Figure 7A; Figure S7I, Supporting Information). This mutation also leads to related NDD symptoms, with the patient exhibiting ID and epileptic symptoms, indicating the importance of the C‐terminal IDR region of SYNCRIP and suggesting that its phase separation characteristics contribute to its normal physiological function.

Next, we aimed to determine whether SYNCRIP possesses phase separation properties by examining both wild‐type and mutant variants of the protein. At 150 mM NaCl and 40 µM EGFP‐SYNCRIP, the formation of green fluorescent protein droplets was clearly visible (Figure 7B,C). However, the mutant proteins, c.858_859del and c.854dupA, formed fewer or no droplets (Figure 7B,C; see Figure S7J,K (Supporting Information) for different NaCl and protein concentrations). The c.1518_1519insC mutant protein, which has a partial loss of the IDR, formed fewer green fluorescent droplets under these conditions (Figure 7B,C; see Figure S7L (Supporting Information) for different NaCl and protein concentrations). The droplet area for the c.858_859del and c.854dupA mutations was significantly reduced, reflecting a marked decrease in fluidity. For c.1518_1519insC, the droplet area did not significantly differ, which could be related to the smaller number of droplets formed by this mutation.

Turbidity is another important characteristic reflecting protein phase separation.^[^ ^45^ ^]^ We measured the turbidity of the purified proteins at 340 nm and detected a lower turbidity for the disease‐causing mutant proteins (Figure 7D). To detect protein exchange kinetics, a characteristic necessary for phase separation, we subsequently used fluorescence recovery after photobleaching (FRAP). EGFP‐SYNCRIP swiftly recovered after bleaching (Figure 7E,F), and the disease‐causing mutants either failed to recover or did so more slowly after FRAP (Figure 7E,F). Similar results were obtained using SNAP‐tagged SYNCRIP in vitro (Figure S7M–O, Supporting Information).

To validate these findings in a cellular context, we transfected NPCs with either EGFP‐SYNCRIP or the disease‐causing mutant constructs. Consistent with our in vitro findings, immunofluorescence analyses showed that EGFP‐SYNCRIP formed prominent cytoplasmic condensates, whereas the disease‐causing mutants c.858_859del and c.854dupA did not form any condensates, and the c.1518_1519insC variant produced fewer condensates (Figure 7I). Given that the c.858_859del and c.854dupA truncations did not form condensates at all, we focused our FRAP experiments on EGFP‐SYNCRIP and c.1518_1519insC. The EGFP‐SYNCRIP exhibited robust fluidity with a fluorescence recovery rate of 54.8 ± 6.5% following photobleaching, whereas the c.1518_1519insC variant displayed markedly reduced fluidity, recovering to only 30.6 ± 4.0% (Figure 7G,H).

These findings indicate that all examined disease‐causing mutations reduce the phase separation capacity of SYNCRIP, even those located outside of recognized functional regions. To confirm that the effects of disease‐causing mutations are not limited to primary NPCs, we performed FRAP analyses in additional cell lines, including HEK‐293T and human neural progenitor cell line (hNPC). The results corroborated our previous observations: EGFP‐SYNCRIP maintained high fluidity, whereas the c.1518_1519insC mutant exhibited significantly reduced fluidity (Figure S8A–D, Supporting Information). Consistently, c.858_859del and c.854dupA mutants failed to form any condensates (Figure S8E,F, Supporting Information).

**Figure 8:**
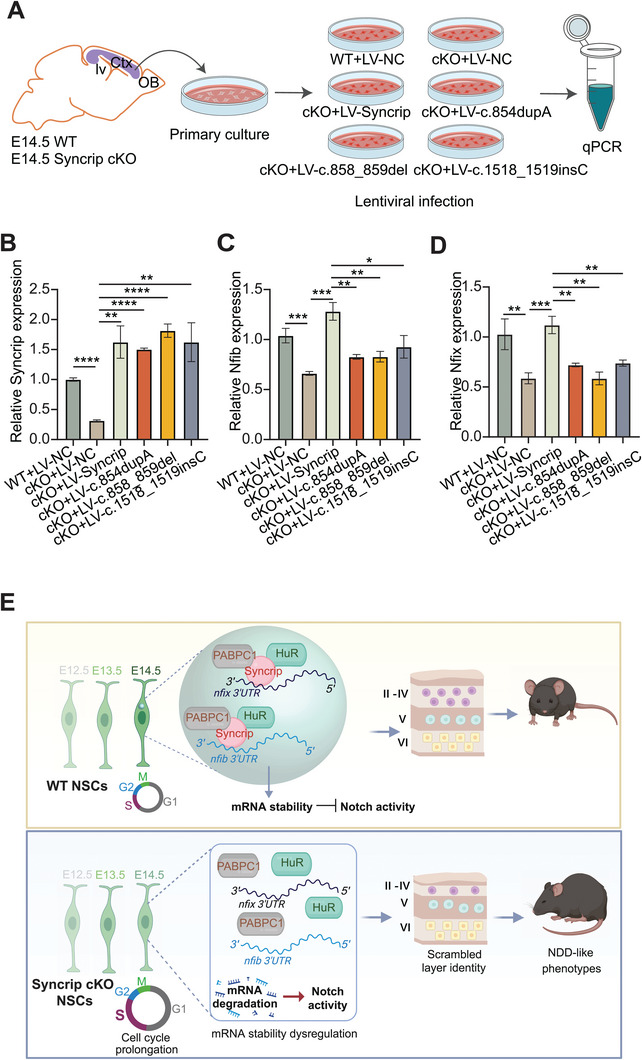
Functional Rescue and Characterization of Syncrip and its Pathogenic Mutations in Primary NPCs from E14.5 Syncrip cKO Mice. A) Schematic of the functional rescue experimental workflow. B–D) Relative mRNA expression of Syncrip B), Nfib C), and Nfix D) in WT+LV‐NC, cKO+LV‐NC, cKO+LV‐Syncrip, cKO+LV‐c.854dupA, cKO+LV‐c.858_859del, and cKO+LV‐c.1518_1519insC groups. Data are presented as the mean ± SEM. Statistical significance was determined using two‐tailed unpaired Student's t‐test. E) Schematic diagram of the role of Syncrip in neocortical development. The data are presented as the mean ± SEM. Statistical significance was determined via an unpaired two‐tailed Student's *t*‐test. *p < 0.05, **p < 0.01, ***p < 0.001, ****p < 0.0001.

We propose that phase separation is critical for SYNCRIP's function. Mutations that diminish this property may prevent SYNCRIP from localizing to P‐bodies and stabilizing downstream RNAs, thereby disrupting NPCs fate and contributing to the pathogenesis of NDD. When we introduced lentiviral vectors encoding EGFP‐tagged SYNCRIP and its disease‐causing mutant proteins into cultured primary mouse NPCs, we observed that SYNCRIP co‐localized with DCP1A (Figure 7I; Movie S1, Supporting Information). In contrast, the c.858_859del and c.854dupA mutants failed to co‐localize with DCP1A, and the c.1518_1519insC mutant showed significantly reduced co‐localization (Figure 7I). We conducted the same experiments in HEK‐293T cells and hNPC cell line and observed consistent results: while EGFP‐SYNCRIP continued to co‐localize with DCP1A (Movies S2 for hNPCs and S3 for HEK‐293T, Supporting Information), the mutant proteins likewise failed to co‐localize with DCP1A. (Figure S8E,F, Supporting Information). In summary, these findings highlight the essential role of SYNCRIP's phase separation capacity in its biological function. Disease‐causing mutants that lose this ability fail to engage with P‐bodies and cannot stabilize their target RNAs, thereby altering NPCs fate and potentially causing NDD. Thus, Syncrip's functional integrity depends on its capacity for phase separation. To further confirm these findings, we attempted to rescue downstream RNA levels in Syncrip cKO primary NPCs derived from the dorsal telencephalon by lentiviral transduction of SYNCRIP or its disease‐causing mutant variants (**Figure** **8**A). QPCR analysis confirmed that lentivirus‐mediated overexpression was successfully achieved in primary NPCs (Figure 8B), for which we designed primers targeting sequences common to both Syncrip and its disease‐causing mutants. We then examined Nfix and Nfib transcript levels. Introduction of Syncrip rescued Nfix and Nfib expression in cKO NPCs, whereas none of the disease‐causing mutants restored their expression (Figure 8C,D). These results underscore that the integrity of the IDR domain is indispensable for the biological function of Syncrip.

## 3 Discussion

### 3.1 The Role of Syncrip in Temporal Patterning of NPCs in Mammalian Neocortex

Our study with Syncrip cKO mice highlights the critical role of Syncrip in neural temporal development and its potential impact on NDD. The behavioral impairments in cKO mice, along with alterations in *Nfib* and *Nfix* mRNA stability and Notch signaling, underscore the intricate molecular pathways through which Syncrip influences the temporal dynamics of RGCs (Figure 8E).

Neocortical development in mammals is a highly temporal process, where RGCs generate specific types of neurons at distinct times. Competence refers to the potential of RGCs to generate specific neuron types within a particular developmental time window, determining their ability to produce specific neurons at specific times.^[^ ^46^ ^]^ As development progresses, the competence of RGCs shifts from generating early neurons to generating late neurons. Previous studies have shown that this temporal regulation depends on the establishment and transition of chromatin modifications such as H3K4me3 and H3K27me3.^[^ ^26^ ^]^ We propose that Syncrip modulates late‐stage neurogenesis in mammals through posttranscriptional regulation. Syncrip is a conserved RBP that controls the differentiation of NPCs by regulating the temporal specification of neuronal fate during Drosophila neurodevelopment.^[^ ^8^ ^]^ Our results indicate that removing Syncrip from the dorsal NPCs of mice leads to defects in the development of the superficial cortical layers. We confirmed that these developmental abnormalities caused by the knockout are due to intrinsic changes in RGCs, with key temporal genes showing a significant shift toward early cortical neurodevelopment. These findings underscore the role of Syncrip in regulating late temporal progression in mammals. Interestingly, this role is analogous to its function in determining late neuronal fate in *Drosophila*, despite the lack of a conserved developmental program between the two species. In *Drosophila*, Syncrip exhibits a gradient of expression from low to high, but no such gradient is observed in the NPCs of the mammalian neocortex. In *Drosophila*, Syncrip primarily functions by inhibiting IMP and Chimo, while in mammals, it is believed to participate in phase separation and regulate signaling pathway activity by controlling the mRNA stability of transcription factors (discussed below).

### 3.2 Impact of Syncrip on Neural Lineage and Neurodevelopmental Disorders

Importantly, the E14.5 to P3 EdU labeling results revealed that Syncrip knockout caused a shift in neuronal lineage toward deeper layers, partially indicating a reverse temporal shift in NPCs lineage, consistent with scRNA‐seq results. This shift led to a trend of increased Ctip2^+^ neurons labeled by E14.5 EdU in Syncrip cKO mice, though these neurons were not located in deeper layers. This finding suggests that the reversal of temporal genes in NPCs does not fully reverse neuronal lineage, which may require more extensive gene regulation or a complete reversal of developmental competence. Nonetheless, Syncrip deletion can partially reverse NPCs lineage. Our results demonstrate that Syncrip is a higher‐order regulatory gene in late‐stage cortical neurogenesis, suggesting a model where the loss of Syncrip leads to a partial loss of late‐stage transcriptional programs and an increase in early temporal core gene expression.

Recent studies have linked Syncrip to protein homeostasis and tumor drug resistance.^[^ ^47^, ^48^ ^]^ Our exploration of molecular mechanisms revealed that Syncrip maintains the activity of key transcription factors through posttranscriptional regulation. Specifically, Syncrip stabilizes transcripts by binding to the 3′ UTRs of *Nfix* and *Nfib*, ensuring their proper translation. This guarantees the proper differentiation of NPCs into superficial neurons. Notably, *Nfix* and *Nfib* are key genes for maintaining stem cell lineage and quiescence, determining cell developmental lineage by regulating chromatin accessibility.^[^ ^49^, ^50^ ^]^ We propose that these transcription factors are crucial for executing the late temporal progression of NPCs. The overexpression of *Nfib* and *Nfix* in vivo can promote the generation of more superficial neurons. Additionally, these two key molecules are crucial for inhibiting Notch activity. Notch signaling plays a central role in maintaining the undifferentiated state of rapidly dividing embryonic NPCs in the mammalian central nervous system.^[^ ^51^ ^]^ Previous studies have shown that Notch activation during cortical development leads to increased stemness of RGCs and a reduction in upper‐layer neurons,^[^ ^52^ ^]^ which was also observed in our research. Our results show that Syncrip directly regulates key molecules and pathways, particularly *Nfix*, *Nfib*, and Notch signaling. These findings enhance our understanding of cortical development and the potential pathogenic mechanisms underlying Syncrip‐related NDDs.

### 3.3 Mechanistic Insights into Syncrip in Neurodevelopment and NDD Pathogenesis

NDDs encompass a group of disorders involving abnormal brain development and function, including ASD, ID, attention deficit hyperactivity disorder (ADHD), and epilepsy.^[^ ^53^ ^]^ The etiology of NDDs is complex, arising from the combined effects of genetic and environmental factors. Mutations in genes involved in RNA metabolism and gene expression regulation, particularly those from the hnRNP family, are common in NDD patients.^[^ ^14^, ^54^ ^]^ In NDD research, Syncrip, a member of the hnRNP family, has been identified as a critical gene associated with NDDs.^[^ ^7^, ^12^, ^13^, ^14^, ^55^ ^]^ Mutations in Syncrip are often *de novo* and significantly enriched in NDD patients. These mutations include likely gene‐disruptive (LGD) and missense mutations, affecting its normal role in neurodevelopment.^[^ ^14^ ^]^ Patients with Syncrip mutations exhibit NDD phenotypes, including developmental delay, ID, ASD, seizures, and structural brain abnormalities.^[^ ^7^, ^12^, ^13^, ^14^, ^55^ ^]^ We utilized Syncrip cKO mice to model the phenotypes observed in patients with Syncrip mutations, including learning and spatial memory impairments. These behavioral abnormalities are closely associated with structural disorganization in the neocortex, especially in the superficial layers. A previous study revealed that ASD patients exhibit synaptic and developmental gene changes in the superficial cortical layers.^[^ ^56^ ^]^ These neocortical defects may disrupt neural circuit formation, leading to memory deficits and learning impairments characteristic of NDDs. LACE‐seq in our study revealed that Syncrip can bind to the mRNA transcripts of many genes associated with neurodevelopment, such as Nfib, Nfix and Zbtb20, which have been confirmed by research to be associated with ID, developmental disorders and ASD.^[^ ^57^, ^58^, ^59^, ^60^, ^61^ ^]^

The hnRNP family is crucial for neurodevelopment as it influences RNA splicing, transport, localization, translation, and degradation. Mutations in hnRNPs can disrupt RNA metabolism during neurodevelopment, leading to NDD.^[^ ^14^ ^]^ Previous studies have suggested a link between Syncrip mutations causing NDDs and phase separation.^[^ ^44^ ^]^ We further highlight the role of Syncrip's phase separation properties in neurodevelopment, linking its RNA stabilization mechanism to NDDs. Syncrip colocalizes with P bodies, membraneless organelles that determine mRNA fate,^[^ ^62^, ^63^ ^]^ and recruits HuR and DCP1A to protect bound RNAs from degradation. However, disease‐causing Syncrip mutations diminish or eliminate the protein's capacity for phase separation. FRAP assays demonstrated that these mutant proteins exhibit markedly reduced fluidity within condensates. Notably, even when nonfunctional regions are truncated, patients still develop NDD phenotypes, underscoring the critical importance of Syncrip's phase separation ability. This finding not only highlights Syncrip's central role in neurodevelopment but also provides fresh insights into the molecular basis of NDDs.

In summary, our research offers new insights into the temporal development programs of RGCs, highlighting Syncrip as a key modulator of late‐stage neurogenesis in the mammalian neocortex through posttranscriptional regulation. These findings deepen our understanding of the molecular basis of NDDs and suggest new avenues for therapeutic intervention. Future studies can explore the therapeutic potential of targeting mRNA stability and signaling pathways affected by Syncrip depletion, offering hope for innovative treatments for NDDs.

## 4 Experimental Section

### Experimental Animals

Mice carrying Syncrip flanked by the loxP allele were generated by Bcgen (Beijing Biocytogen Co., Ltd.) and mated with Nestin‐Cre (Jackson Laboratories, stock number 003771) and Emx1‐Cre (Jackson Laboratories, stock number 005628) mice to generate various Syncrip cKO mutants, which were in the C57BL/6 background. The mice were maintained at the Peking Union Medical College Animal Center. All procedures were authorized by the Institutional Animal Care and Use Committee of the Chinese Academy of Medical Sciences and Peking Union Medical College (ACUC‐A01‐2023‐028). The noon of the day when the vaginal plug was found was counted as embryo (E) day 0.5.

### Tissue Section

The pregnant mice were intraperitoneally anesthetized with 0.7% w/v sodium pentobarbital (0.01 ml g^−1^ body weight) in 0.9% saline. Embryonic mouse brains were then dissected out in cold PBS. After being anesthetized with the same agent, P3/P7 mice were subjected to transcardial perfusion with 4% PFA‐PBS. Both embryonic and P3/P7 brain samples were fixed in 4% PFA for 24 h, followed by dehydration in 25% sucrose for another 24 h. The brains were subsequently embedded in O.C.T. (SAKURA) and stored at −80 °C. They were then cut into 16 µm thick sections using a Leica CM1950 cryostat.

### Immunofluorescence

The tissue sections were desiccated at 50 °C for 30 min and then washed in 1× PBS for 5 min. For heat‐mediated antigen retrieval, the sections were incubated with 10 mM sodium citrate buffer (pH 6.0) at 95 °C for 20 min, followed by natural cooling. For BrdU staining, the sections were treated with 2 N HCl for 10 min at 30 °C and subsequently for 20 min at room temperature. The sections were subsequently incubated with 5% sheep serum (1× PBS, 0.3% Triton X–100) for 1 h at room temperature. The tissue sections were incubated overnight at 4 °C with primary antibodies, prepared in buffer (1 × PBS, 0.3% Triton X‐100). The primary antibody mixture was removed, and the sections were washed three times in PBS for 5 min each. The secondary antibodies were diluted with buffer solution at a ratio of 1:800 and incubated with the tissue sections for 1 h at room temperature. After three rinses in PBS, the nuclei were stained with 4′,6‐diamidino‐2‐phenylindole (DAPI) and then mounted. The tissue slices were observed and photographed with a Leica system (TCS‐SP8 STED 3X).

### In Situ Hybridization

ISH was performed as previously described.^[^ ^4^ ^]^ Briefly, the sections were sequentially incubated with 4% paraformaldehyde in 0.1 M phosphate buffer, followed by 2 µg ml^−1^ proteinase K and then 0.25% acetic acid in 0.1 M triethanolamine, with two PBS washes between each step. Prehybridization involved a 1 h incubation at room temperature in a hybridization mixture (50% formamide, 5x SSC, 5x Denhardt's solution, 250 µg ml^−1^ yeast RNA, 500 µg ml^−1^ herring sperm DNA), followed by overnight hybridization at 65 °C with 0.5 µg ml^−1^ DIG‐labeled RNA probes in the same buffer. The sections were then washed three times in 0.2X SSC at 65 °C and twice at room temperature in B1 buffer (0.1 M Tris‐HCl pH 7.5, 0.15 M NaCl). Blocking was performed for 1 h at room temperature with B2 buffer (0.1 M Tris‐HCl pH 7.5, 0.15 M NaCl, and 10% sheep serum), followed by overnight incubation at 4 °C with an alkaline phosphatase‐conjugated anti‐digoxigenin antibody (1:3000). After three washes in B1 buffer at room temperature, the sections were equilibrated in B3 buffer (0.1 M Tris‐HCl pH 9.5, 0.1 M NaCl, 0.05 M MgCl2, 0.1% Tween 20) twice. Color development occurred at room temperature in B4 buffer (0.1 M Tris‐HCl pH 9.5, 0.1 M NaCl, 0.05 M MgCl2, 0.1% Tween 20, 2% NBT/BCIP) and was stopped with double distilled water upon reaching the desired intensity. Finally, the sections were prepared for microscopy with Mowiol mounting medium.

### TUNEL assay and 5‐Ethynyl‐2′‐deoxyuridine (EdU) staining

TUNEL staining was conducted on frozen tissue sections using an in situ Cell Death Detection Kit (Roche, 11 684 817 910). Cell proliferation was assessed using the Click‐iT Plus EdU Cell Proliferation Kit for Imaging (Invitrogen, CC10640) according to the manufacturer's protocols. In brief, following standard immunofluorescence staining, the sections were treated with the EdU working solution for 1 h at room temperature in the dark prior to mounting.

### NPCs Culture

Mouse NPCs from E14.5 mice were obtained by dissecting the lateral ventricles, followed by digestion into a single‐cell suspension with Accutase (Sigma). NPCs were maintained in DMEM/F12 proliferation medium supplemented with 2% B27 supplement, 20 ng ml^−1^ EGF, 20 ng ml^−1^ bFGF, 1% GlutaMAX supplement and 0.2% BSA. After culture in vitro for three generations, NPCs were subjected to Western blotting, RT‒qPCR and LACE‒seq.

### Western Blot

E14.5 and P7 forebrains were swiftly isolated and lysed in TNTE buffer (50 mM Tris‐HCl, 150 mM NaCl, 1 mM EDTA, 1 mM Na_3_VO_4_, 25 mM NaF, 10 mM Na_4_P_2_O_7_⋅10H_2_O, 0.5% Triton X‐100 and protease inhibitors). The lysates were incubated for 30 min on ice and centrifuged at 12 000 rpm for 30 min at 4 °C. Proteins were separated on 10%–12% SDS‒PAGE gels and transferred to nitrocellulose membranes. Prior to incubation with primary antibodies overnight, the membranes were blocked with 5% nonfat milk dissolved in TBS‐Tween 20 (0.05%) for 1 h at room temperature.

### scRNA‐seq

Brain dorsal cortical tissues were carefully dissected from one E14.5 wild‐type (WT) embryo and one E14.5 Syncrip cKO embryo obtained from the same pregnant mouse. Each sample was then subjected to single‐cell RNA sequencing (scRNA‐seq) library preparation and subsequent analysis. Single‐cell library construction was performed in accordance with the protocol provided by the 10x Genomics single‐cell 3′ Library and Gel Bead Kit V3.1 (10x Genomics, 1 000 075). A Chromium Single Cell Controller (10x Genomics) was used to process cell suspensions at a density of 300–600 viable cells per µL, creating single‐cell gel beads within an emulsion. Single cells were then resuspended in PBS supplemented with 0.04% BSA. Approximately 6000 cells were loaded per channel, resulting in the recovery of ≈3000 cells. The captured cells underwent lysis, releasing RNA that was barcoded via reverse transcription within each GEM. This process was facilitated via an S1000TM Touch Thermal Cycler (Bio‐Rad, USA), in which the GEMs were set at 53 °C for 45 min, then at 85 °C for 5 min, and held at 4 °C. The complementary DNA (cDNA) library was subsequently produced, amplified, and assessed for quality control analysis via an Agilent 4200 instrument. Finally, the single‐cell RNA‐seq libraries were sequenced on an Illumina NovaSeq 6000 system, ensuring a minimum sequencing depth of 100 000 reads per cell (performed by CapitalBio Technology, Beijing).

The Seurat R package was used to process and analyze the scRNA‐seq data from the E14.5 mouse neocortex. Initially, a single‐cell object was created using the CreateSeuratObject, with min.cells = 3 and min.features = 200. The cells with more than 10% mitochondrial gene content were subsequently filtered out. We performed neighbor identification and clustering analysis of cells via FindNeighbors and FindClusters, with the clustering resolution set to 0.8. Arlotta single‐cell data were mapped via FindTransferAnchors, and low‐quality cells were removed and repopulated. Cell cycle scores were computed via CellCycle scoring using known markers of brain cell types.

Potency score and potency category predictions were obtained via developmental potential prediction (CytoTRACE 2), an interpretable deep learning framework from scRNA‐seq data.^[^ ^29^ ^]^

For birthdate score calculation, it was used Wu's method,^[^ ^26^ ^]^ which involves regression modeling using published data and selecting genes on the basis of the linear weights of the model. For the maturation score calculation, it was adopted Matthieu X. Moreau's method,^[^ ^27^ ^]^ which also uses Telly's scRNA‐seq data^[^ ^6^ ^]^ to obtain the dorsal NSC data from E12–E15.

For the analysis of transcription factor activity, we utilized pySCENIC to analyze the cell data of mouse RGCs and IPs. Specifically, we used SCENIC to generate regulons, extracted the non‐duplicated AUC data, normalized it, and then fitted a linear model for comparative analysis.^[^ ^70^ ^]^

### RT‒qPCR

Total RNA from E14.5 NPCs from both control and Syncrip cKO mice was isolated using TRIzol reagent (Invitrogen). cDNA was synthesized using a Reverse Transcriptase Kit (TaKaRa). Quantitative RT‒PCR was conducted with a SYBR Green‐containing kit (TaKaRa). The primer sequences could be found in Table S1, with β‐actin serving as the internal control.

### mRNA Stability

NPCs from E14.5 control and Syncrip cKO mice at three generations were plated on Matrigel and treated with actinomycin D. RNA was extracted at 0, 3, 9, and 12 h post treatment. The stability of the nfib and nfix mRNA transcripts was assessed via qPCR.

### In Utero Electroporation

In utero electroporation (IUE) was performed as previously described.^[^ ^4^ ^]^ Pregnant mice were anesthetized with pentobarbital sodium (0.7 mg g^−1^). E13.5 embryonic brains received five 30 V pulses (50 ms on/950 ms interval) via 7‐mm platinum electrodes using a BTX‐ECM830 electroporator (Harvard Apparatus). For the rescue experiments, pLL3.7‐scr, pCIG, pLL3.7‐shSyncrip, pCIG‐NFIB OE, and pCIG‐NFIX OE were electroporated at 2.5 µg µl^−1^.

### Immunoprecipitation

HEK‐293T cells were transfected with pcDNA3.1‐Syncrip for 48 hours and then lysed using Pierce IP Lysis/Wash Buffer containing protease inhibitors at 4 °C for 30 min. The lysate was centrifuged at 12 000 rpm for 20 min at 4 °C, and the supernatant was collected. Five hundred micrograms of total protein were incubated with anti‐FLAG magnetic beads overnight at 4 °C to immunoprecipitate FLAG‐Syncrip. The next day, the beads were washed three times with Pierce IP Lysis/Wash Buffer. Subsequently, 6× SDS‐PAGE loading buffer was added, and the immunoprecipitated proteins were denatured at 98 °C for 10 min. The samples were then analyzed by Western blotting using anti‐HuR and anti‐PABPC1 primary antibodies.

### LACE‐seq

LACE‐seq experiments were conducted following the established protocol.^[^ ^32^ ^]^ LACE‐seq was performed on NPCs isolated from E14.5 embryos and cultured, and LACE‐seq was performed as described previously. First, the samples were cross‐linked twice on ice with UV‐C, followed by RNA immunoprecipitation with anti‐Syncrip and mouse IgG, after which the captured RNA was fragmented using MNase, and RNA adapters were added after dephosphorylation. Next, reverse transcription was performed, the first strand of cDNA was captured with streptavidin affinity magnetic beads, cDNA adapters were added, and pre‐PCR, IVT, RT, PCR barcoding, and deep sequencing were sequentially performed.

The adapter sequence at the 3′ end of the original reads and the adapter sequence at the 5′ end were removed using Cutadapt:^[^ ^64^ ^]^ ‐a ATCTCGTATGCCGTCTT ‐a CTGCTCGTATGCCGTCTTCTGCTTG ‐n 10 ‐m 22. Clean reads were compared to mouse pre‐rRNA using a Bowtie,^[^ ^65^ ^]^ and the remaining unmapped reads were subsequently compared to the mouse (mm10) reference genome. For LACE‐seq data mapping, two mismatches were allowed (Bowtie parameters: ‐v 2 ‐m 10 ‐best ‐strata; ‐v 2 ‐k 10 ‐best ‐strata). Peaks were identified by Piranha (http://smithlabresearch.org/software/piranha/) with the following parameters: ‐s ‐b 20 ‐p 0.01. Peaks without an IgG signal was selected for further analysis.

### RNA‐seq

Total RNA from E14.5 NPCs from both control and Syncrip cKO mice was isolated using TRIzol reagent (Invitrogen) and evaluated with an Agilent 2100 Bioanalyzer (Agilent Technologies, Santa Clara, CA, USA) and a Qubit Fluorometer (Invitrogen). RNA‐seq libraries were generated and sequenced by CapitalBio Technology (Beijing, China). The sequencing libraries were constructed using the NEBNext Ultra RNA Library Prep Kit for Illumina (NEB). The final libraries were quantified using KAPA Library Quantification Kit (KAPA Biosystems, South Africa) and an Agilent 2100 Bioanalyzer. After RT‒qPCR validation, the libraries were subjected to paired‐end sequencing with paired‐end 150‐base pair reading lengths on an Illumina NovaSeq sequencer (Illumina).

The sequencing quality was assessed with FastQC (v0.11.5),^[^ ^66^ ^]^ and low‐quality data were filtered out via NGSQC (v2.3.3).^[^ ^67^ ^]^ The clean reads were then aligned to the mouse UCSC mm10 genome using HISAT2^[^ ^68^ ^]^ with default parameters. The processed reads from each sample were aligned against the reference genome via HISAT2. DESeq2^[^ ^69^ ^]^ was used to identify differentially expressed genes between samples.

Gene set enrichment analysis (GSEA) in this study was performed using clusterProfiler. To identify significantly enriched Gene Ontology (GO) terms (FDR <0.05), the Database for Annotation, Visualization and Integrated Discovery was used.

### Behavior Tests

Approximately 12–16‐week‐old male mice were used for testing. The mice were housed in standard cages with 4–5 animals each, with access to water and food. All of the behavioral tests were performed in a blinded manner. The behavioral tests included the Morris water maze, Y maze, open field, elevated plus maze, and three‐chamber tests.

*Morris water maze*: The mice were introduced to a black plastic circular water‐filled tank, which was 120 cm in diameter and 50 cm in height, with nonreflective internal surfaces and ample visual cues. Two main axial lines were drawn on the bottom of the tank, each of which cut the maze vertically in a “+” shape. The endpoints of each line are labeled with four cardinal points: east, south, west, and north. To hide the platform, a white, nontoxic food additive was added to the water in the tank. A 10‐cm‐diameter circular Plexiglas platform was immersed in the water, located in the center of the southeast quadrant, with the top surface of the platform 1 cm below the water surface. Each mouse had a different starting point each day, and each mouse was subjected to 3 trials per day, each 60 min apart, for 5 days. Each trial lasted 1 min, and the trial ended when the mouse swam to and stayed on the hidden platform for 5 s. The mice were not allowed to stay on the platform any longer than 5 s. The time it took the mice to find the platform was called latency, and for each mouse, the latency time of the 3 trials was averaged and recorded as a result. On day six, the mice were subjected to a 60 s probe trial without the platform to test memory retention. The mice started the trial from the northwest, the number of platform traversals was counted, and the swimming path was recorded and analyzed via a Noldus.

*Open field test*: The mice were gently placed along the wall in an open‐field device measuring 50 cm in length, width, and height and allowed to explore freely for 5 min. The movements of the mice were recorded by a video camera and analyzed with Noldus.

*Y maze*: The Y‐maze was used to study cognitive ability and spatial working memory. This device consists of three radiating arms that were arranged at equal angles (120°). At the beginning of the trial, the experimental mice were placed in one of the fixed arms and allowed to move freely in the maze for 5 min. Efficient behavior was defined as continuous access to all three arms in overlapping sets of three choices.

*Elevated‐plus maze*: The elevated plus maze was located 50 cm above the ground and consisted of two open arms and two closed arms. Test mice were placed in the center of the maze. The time spent and distance traveled in different arms during 5 min of free exploration were subsequently measured.

*Three‐chamber social test*: This assay consisted of three chambers with two cages in the left or right chamber, each separated by a door. The experiment was divided into three phases. The first phase allowed the test mice to freely explore all three chambers for 10 min to acclimatize. The second phase was the socialization test. An age‐matched novel mouse was put into one cage while the other cage was empty. The test mouse was gently placed into the center and allowed to explore the three chambers for 5 min. In the third stage, another novel mouse was placed into the empty cage, and the test mouse was allowed to explore freely for 5 min. The time spent in each chamber was measured.

### Luciferase Assays

HEK‐293ET cells in a 24‐well plate were cotransfected with the reporter plasmid, Renilla luciferase expression vector (pRL‐TK, serving as a normalization control), and various effector plasmids via PEI. The cells were collected after 72 h, and the Dual‐Luciferase Reporter Assay System from Promega was used to measure the activity levels of both firefly and Renilla luciferase within the same sample. The activity of each reporter was evaluated over the course of three separate experiments, with each experiment consisting of four replicates.

### Purification of Recombinant SYNCRIP Proteins

To purify recombinant proteins for phase separation assays, BL21 (DE3) receptor cells were transformed with plasmids encoding Syncrip and its pathogenic mutants and allowed to grow at 37 °C, and protein expression was induced with isopropyl‐β‐D‐thiogalactopyranoside for 20 h at 20 °C. Bacteria were fragmented by sonication after centrifugation and resuspension. The induced expressed proteins were incubated with Ni‐nitrilotriacetic acid (NTA) resin (Qiagen, #30 210) and incubated with 50 ml of wash buffer I [150 mM NaCl, 20 mM Tris‐HCl, 5% glycerol, 5 mM imidazole (pH 8.0)], 50 ml of wash buffer II [150 mM NaCl, 20 mM Tris‐HCl, 5% glycerol, 10 mM imidazole (pH 8.0)] and 25 ml of wash buffer III [150 mM NaCl, 20 mM Tris‐HCl, 5% glycerol, 15 mM imidazole (pH 8.0)] to wash the column, and the target protein was eluted with elution buffer IV [150 mM NaCl, 20 mM Tris‐HCl, 5% glycerol and 300 mM imidazole]. To remove imidazole and adjust the protein to the desired working concentration, the eluted protein fractions were passed through Pierce Protein Concentrators PES (Thermo Fisher, 88 525 and 88 531), which effectively reduce small molecule contaminants like imidazole. The final protein preparations were verified by SDS‒PAGE to ensure purity and integrity.

### In Vitro Phase Separation Assays

Purified Syncrip and its mutant proteins were diluted with phase separation buffer containing different concentrations of NaCl (37.5 to 300 mM), 20 mM Tris‐HCl (pH 8.0), 5% glycerol, and 1 mM DTT to achieve final concentrations ranging from 5 to 40 µM, and 10% PEG8000 was added. After protein phase separation at different protein and salt concentrations, the reaction was carried out at room temperature for 30 min, and images of the phase separation condensate were taken using a Leica Stellar confocal microscope.

### Fluorescence Recovery after Photobleaching (FRAP)

To determine the dynamic mobility of the Syncrip proteins, we photographed them in FRAP mode with a Leica Stellar confocal microscope. Phase‐separated droplets of Syncrip and its pathogenic mutant proteins were prepared in buffer containing 150 mM NaCl, and the droplet centers were photobleached with 488 nm laser pulses (100% intensity). For FRAP data analysis, Leica Application Suite X and GraphPad Prism 9.0.0 software were used to calibrate the recovery of fluorescence intensity within the bleached droplets.

For cellular FRAP, we transfected primary mouse NPCs, hNPC cell line, and HEK293T cell line with EGFP‐Syncrip constructs, including the c.1518_1519insC disease‐causing mutant, and performed ten independent photobleaching events on cytoplasmic Syncrip‐positive condensates. It was then tracked the fluorescence recovery, using Leica Application Suite X and GraphPad Prism 9.0.0 to quantify recovery curves and extract kinetic parameters.

### Lentivirus Packaging and Transduction

Following the manufacturer's instructions, Lenti‐X 293T cells were co‐transfected with the transfer plasmid, packaging plasmid (psPAX2), and envelope plasmid (pMD2.G) using transfection reagent (YEASEN, 40802ES03) to produce lentiviral vectors expressing the target gene or its mutant variants. Viral supernatants were collected at 24, 48, and 72 h post‐transfection, filtered through a 0.45 µm membrane, and then concentrated using a lentivirus concentration solution (Takara) according to the supplier's protocol. The resulting lentivirus preparations were subsequently used to transduce primary NPCs, which were maintained under standard culture conditions.

### SNAP‐tag Labeling of Purified Protein

Purified SNAP‐tagged SYNCRIP protein was incubated with SNAP‐Surface Alexa Fluor 647 substrate (NEB, S9136) in PBS (1X) with 1 mM DTT at 37 °C in the dark for 30 min, following the manufacturer's instructions. After labeling, the protein was visualized directly by confocal microscopy.

## Conflict of Interest

The authors declare no conflict of interest.

## Author Contributions

All authors participated in the scientific discussion. X.P. and P.S. conceived the research. X.P., P.S., J.W., H.Y. and X.D. designed the experiments. J.W., H.Y., X.D., B.Y. and L.H. carried out the experimental studies and analyses. Y.X. guided the construction of LACE‐seq library. H.Y. performed bioinformatics analysis. J.W., H.Y. and P.S. wrote the manuscript. X.P., P.S., B.Q. and Y.X. revised the manuscript. All authors commented on the manuscript.

## Supporting information



Supporting Information

Supplemental Movie 1 (3D reconstruction of E14.5 mouse primary NPCs)

Supplemental Movie 2 (3D reconstruction of hNPC cell line)

Supplemental Movie 3 (3D reconstruction of HEK‐293T cell line)

## Data Availability

The data that support the findings of this study are available from the corresponding author upon reasonable request. All data generated in this study have been deposited in the Gene Expression Omnibus (GEO) under accession number GSE285581.
