# Supplementary material for: Posttranscriptional Control of Neural Progenitors Temporal Dynamics During Neocortical Development by Syncrip
Source: Adv Sci (Weinh). 2025 Jan 7;12(8):2411732. doi: 10.1002/advs.202411732 (PMC11848603; doi:10.1002/advs.202411732)
Supplement: Supplementary file 1 — Supporting Information [file ADVS-12-2411732-s004.docx]

**Supporting information**

**Posttranscriptional Control of Neural Progenitors Temporal Dynamics during Neocortical Development by Syncrip**

*Jiarui Wu, Haoyang Yu, Xinyi Dou, Bin Yin, Lin Hou, Yuanchao Xue, Boqin Qiang, Pengcheng Shu, Xiaozhong Peng*

**This file includes:**

Supplementary Table S1- Supplementary Table S2

Supplementary Figure S1-S8

Supplementary Movie 1-3

**Supplementary Table S1. Primary Antibodies Used in This Paper.**

| Antibodies | Experiment | Supplier | Item number |
| --- | --- | --- | --- |
| Rabbit anti-Syncrip | IF/WB | Solarbio | Cat# K000436P |
| Rabbit anti-Pax6 | IF | Convance | Cat# prb-278p |
| Mouse anti-Pax6 | IF | BD | Cat#561462 |
| Rabbit anti-Tbr2 | IF | Abcam | Cat# ab23345 |
| Rabbit anti-Cux1 | IF | Santa Cruz | Cat# sc13024 |
| Rat anti-Ctip2 | IF | Abcam | Cat# ab18465 |
| Mouse anti-Tle4 | IF | Santa Cruz | Cat# sc365406 |
| Rabbit anti-NeuroD2 | IF | Abcam | Cat# ab104430 |
| Rabbit anti-Calbindin | IF | Oasis Biofarm | Cat# OB-PRB052-01 |
| Rabbit anti-Brn2 | IF | Santa Cruz | Cat# SC6029 |
| Rabbit anti-Ki67 | IF | Abcam | Cat# ab16667 |
| Rat anti-BrdU | IF | Abcam | Cat# ab6326 |
| Rabbit anti-Sox5 | IF | Abcam | Cat# ab26041 |
| Rabbit anti-Aldh1l1 | IF | Oasis Biofarm | Cat# OB-PRB001-01 |
| Rabbit anti-Syncrip | WB | Thermo Fisher | Cat# PA5-99420 |
| Rabbit anti-Nfib | WB | Sigma | Cat# HPA003956 |
| Mouse anti-Nfix | WB | Proteintech | Cat#67983-1 |
| Mouse anti-HuR | WB | Santa Cruz | Cat# sc-5261 |
| Rabbit anti-NICD | WB | Cell Signaling Technology | Cat#2421 |
| Mouse anti-Syncrip | LACE-seq | Millipore | Cat#05-1517 |
| Mouse IgG | LACE-seq | Santa Cruz | Cat#sc2025 |
| Mouse anti-β-actin | WB | Sigma | Cat# A5441 |

**Supplementary Table S2. Primers Used in This Paper.**

| Syncrip-genotyping-F: | AACTGCTGCATGTGGCAGG |
| --- | --- |
| Syncrip-genotyping-R: | CATGTTCTGTAGCCATGTTTCC |
| Cre-genotyping-F: | TCGATGCAACGAGTGATGAG |
| Cre-genotyping-R: | TTCGGCTATACGTAACAGGG |
| mNfib-qpcr-F: | CAGGAGCAAGATTCTGGAC |
| mNfib-qpcr-R: | GGGTGTTCTGGATACTCTCAC |
| mNfix-qpcr-F: | CTGGCTTACTTTGTCCACACT |
| mNfix-qpcr-R: | CCAGCTCTGTCACATTCCAGA |
| mActb-qpcr-F: | TGTCCACCTTCCAGCAGATGT |
| mActb-qpcr-R: | AGCTCAGTAACAGTCCGCCTAGA |
| Syncrip-qpcr-F | TGGAAAGAACAGGCTACACACTTG |
| Syncrip-qpcr-R | CTCAGTGCCAACAGAAGGCTG |

**
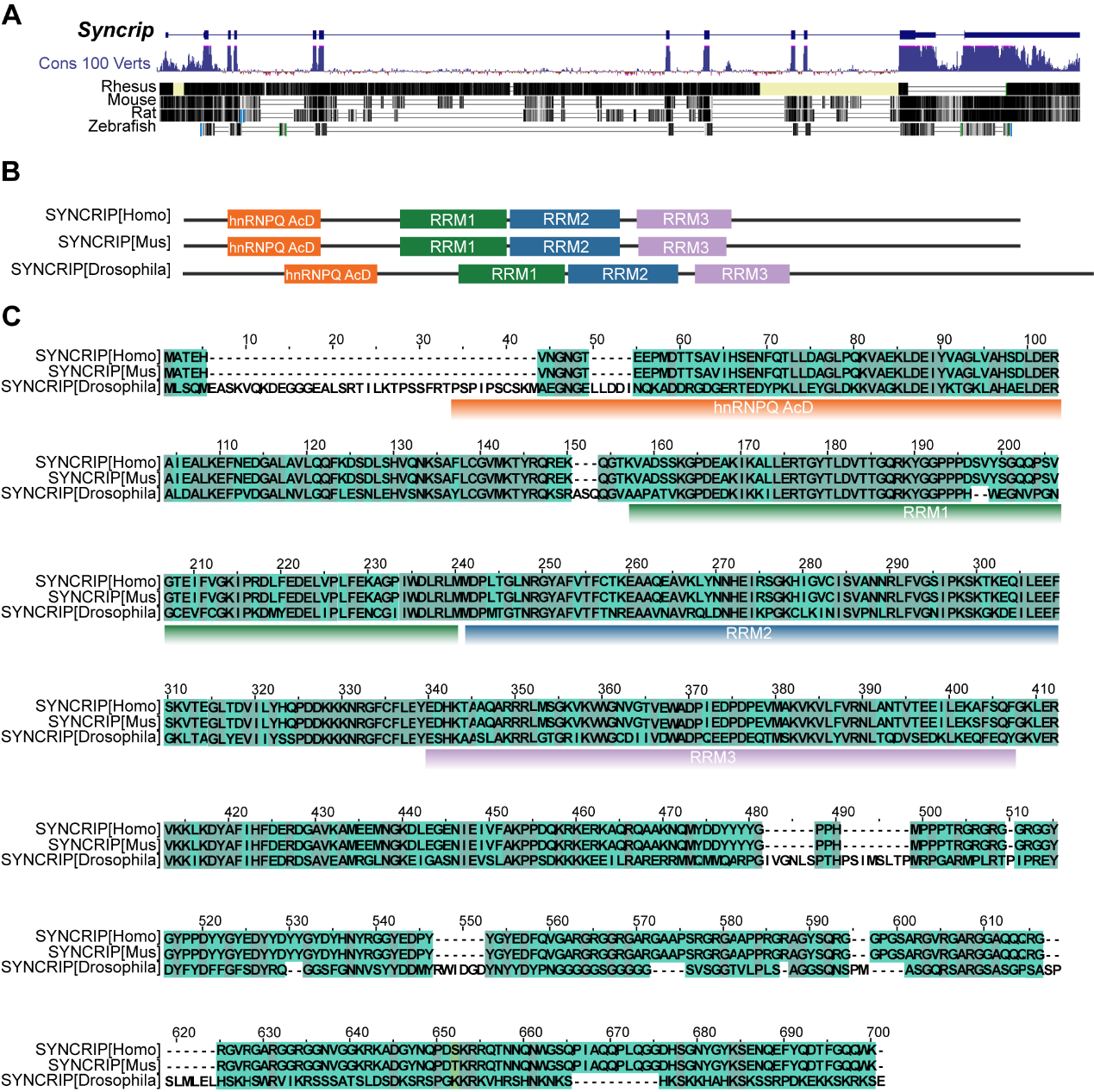
**

**Supplemental Figure S1. SYNCRIP: Conservation Across Multiple Species.**

1. Comparative genomic conservation of Syncrip across rhesus, mouse, rat, and zebrafish. Data were obtained from the UCSC Genome Browser (http://genome.ucsc.edu).
2. Comparison of SYNCRIP protein domains across human, mouse, and fly.
3. Comparison of SYNCRIP protein amino acid sequences across human, mouse, and fly.

**
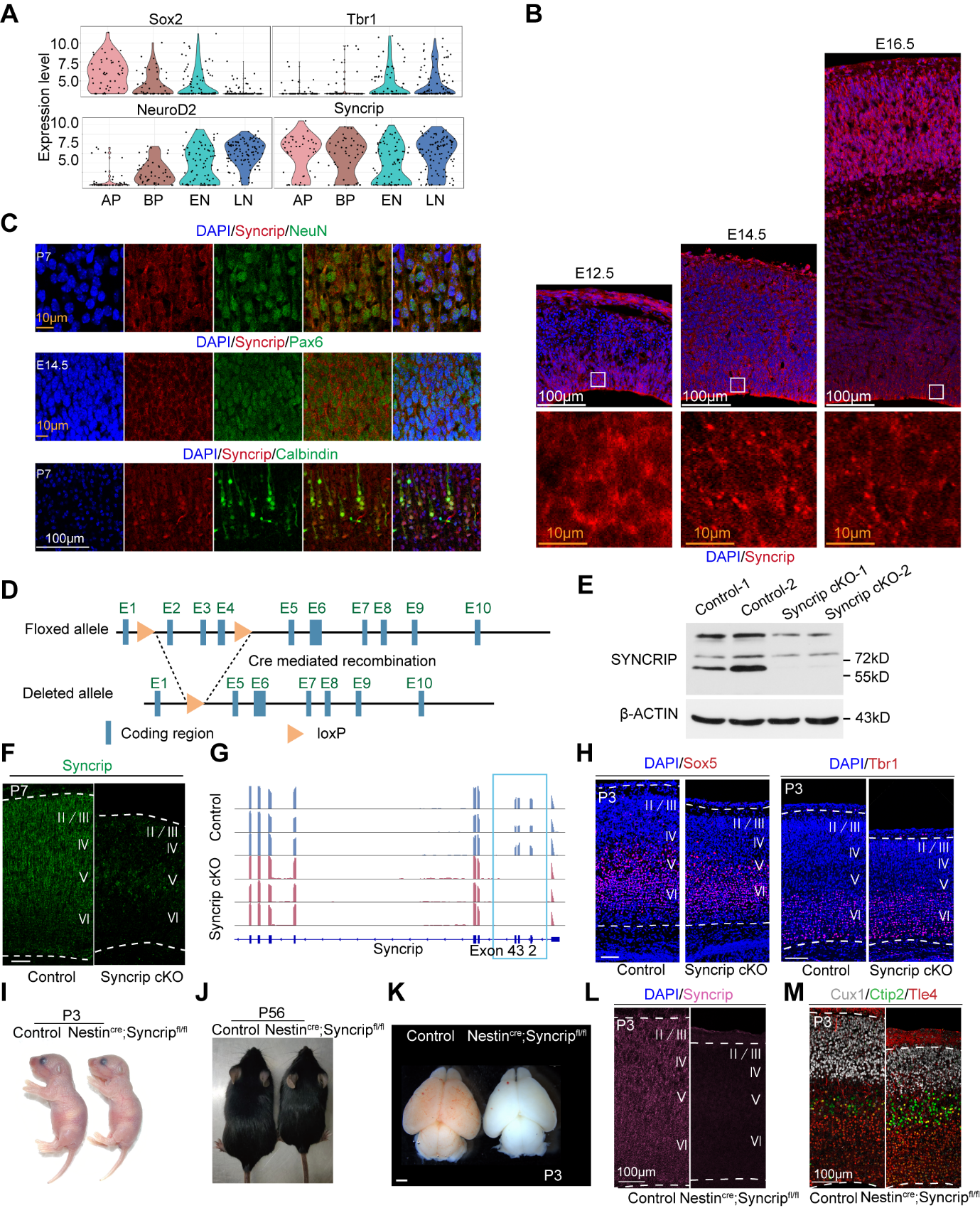
**

**Supplemental Figure S2. Syncrip Expression and Impact on Neocortical Development.**

1. Expression of Syncrip on the basis of a published scRNA-seq atlas of the developing neocortex. The expression profiles of the chosen genes are shown as violin plots. AP: apical progenitors; BP: basal progenitors; EN: early neurons; LN: late neurons.
2. Immunofluorescence (IF) staining of Syncrip in coronal sections of the E12.5, E14.5 and E16.5 neocortical regions. Nuclei were labeled with DAPI (blue).
3. Double IF staining of Syncrip with the pyramidal neuron marker NeuN, the RGC marker Pax6 and the interneuron marker calbindin in coronal sections of the E14.5 and P7 neocortex. Nuclei were labeled with DAPI (blue).
4. Schematic representation of the Syncrip-targeted mouse generation strategy. Exons 2, 3, and 4 are flanked by loxP sites and will be excised upon crossing with Cre recombinase-expressing mice.
5. Western blot of SYNCRIP and β-actin in P7 control and Syncrip cKO neocortical extracts.
6. IF staining of Syncrip in coronal sections of the control (left) and Syncrip cKO (right) P7 neocortex.
7. The RNA-seq tracks of E14.5 control and Syncrip cKO neocortical neurospheres showed deletion of exons 2, 3, and 4 (blue box).
8. IF staining of the cortical PN markers Sox5 and Tbr1 in coronal sections of control (left) and Syncrip cKO (right) P3 neocortices.
9. Comparison of the body sizes of Nestin^cre^;Syncrip^fl/fl^ mice and control mice at P3.
10. Comparison of the body sizes of Nestin^cre^;Syncrip^fl/fl^ mice and control mice at P56.
11. Brain dissections revealed that Nestin^cre^;Syncrip^fl/fl^ mice presented significant defects in the dorsal forebrain at P3.
12. IF staining of Syncrip in coronal sections of the control (left) and Nestin^cre^;Syncrip^fl/fl^ (right) P3 neocortex.
13. IF staining of the cortical PN markers Cux1, Ctip2 and Tle4 in coronal sections of the control (left) and Nestin^cre^;Syncrip^fl/fl^ (right) P3 neocortex.

Scale bars, 2 mm (k), 100 μm (white, b, c, f, h, l, m), and 10 μm (orange, b, c).

**
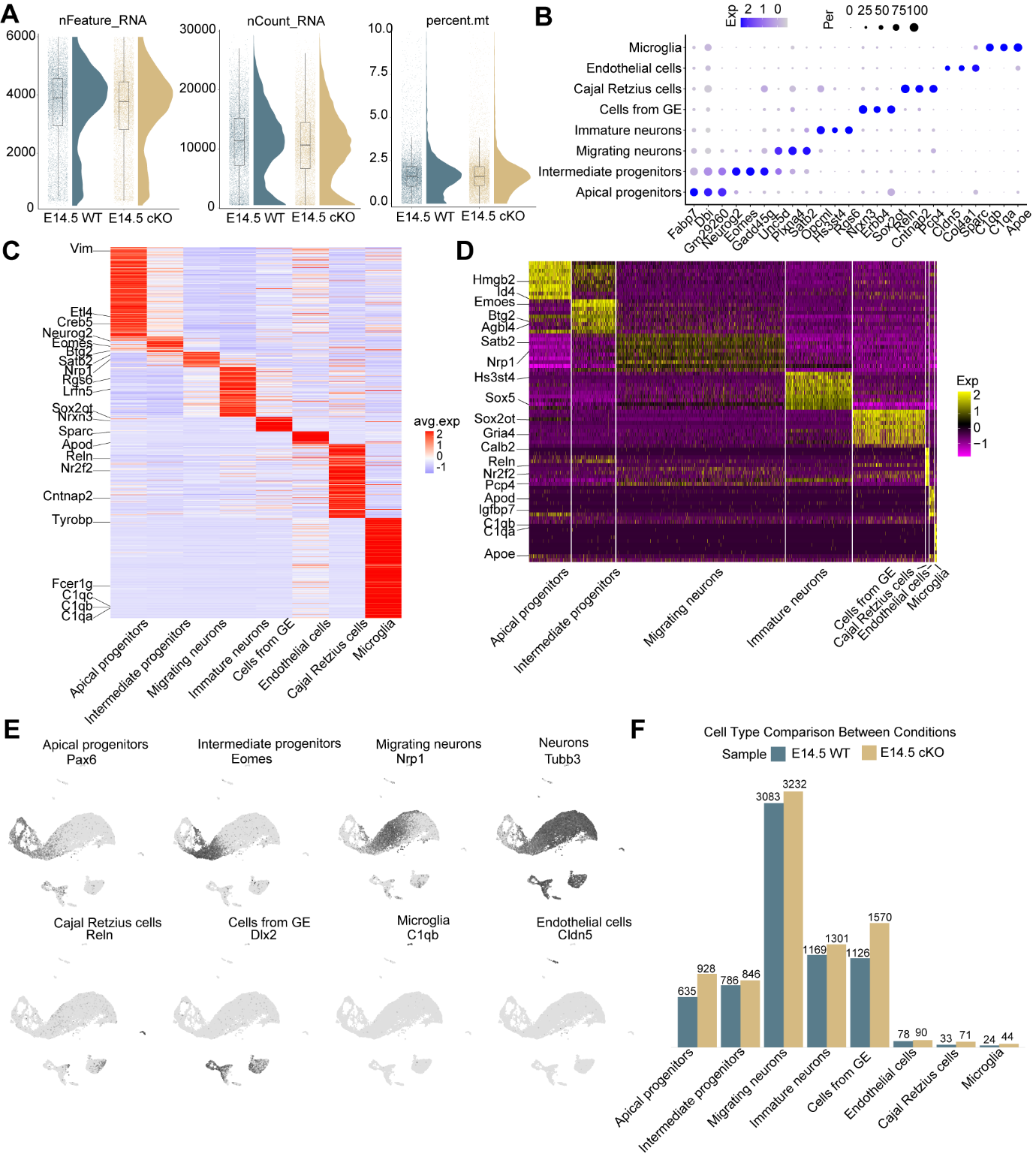
**

**Supplemental Figure S3. Molecular Signatures and Cell Types Classified via scRNA-seq from Control and Syncrip-deficient E14.5 Neocortical Samples.**

1. Number of genes, number of mRNA molecules (counts) and percentage of mitochondrial counts per cell in the control and Syncrip cKO E14.5 neocortex.
2. The eight cell types identified using the 10× Genomics platform was characterized and annotated, and a dot plot was generated to display the marker gene expression profiles for each cell type.
3. Heatmap showing the varying expression of gene sets in different clusters.
4. Gene signatures for all cell types identified in the control and Syncrip cKO E14.5 neocortical regions. The top 10 DEGs for each cell type are presented.
5. Expression of canonical marker genes for selected cell types in the UMAP visualization of the combined scRNA-seq course.
6. Quantification of cell types identified from scRNA-seq analysis of the E14.5 cortex.


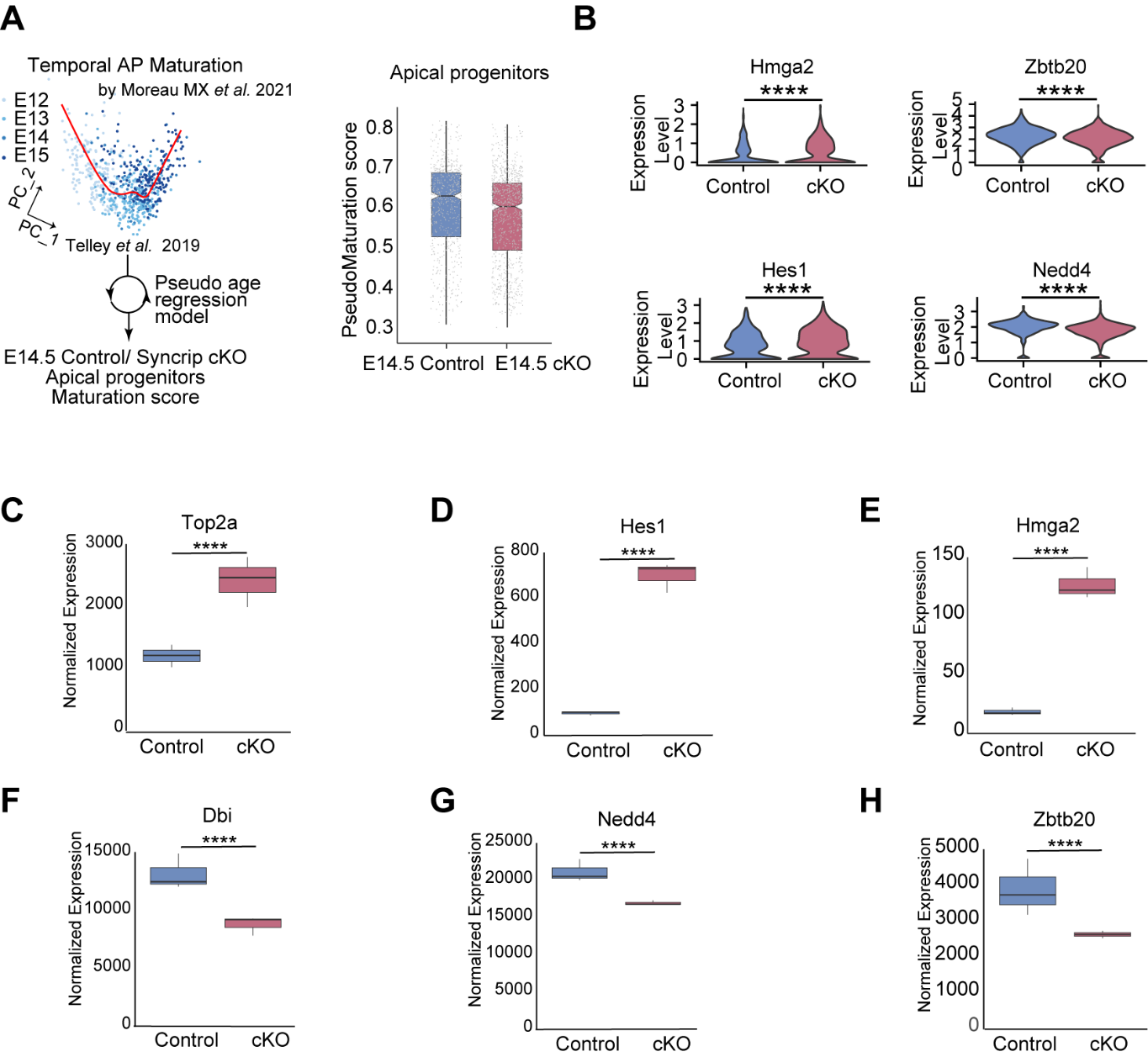


**Supplemental Figure S4. Temporal Gene Expression in E14.5 Syncrip cKO NPCs.**

1. By utilizing the time series dataset from Telley et al. (2019) for maturation score calculation, a regression model was trained to predict these scores for control and Syncrip cKO APs.
2. Violin plots from the scRNA-seq analysis showing differential expression of Hmga2, Hes1, Zbtb20, and Nedd4 in E14.5 Syncrip cKO APs compared with controls.

(C-H) Boxplots showing the normalized expression levels of selected genes in WT and cKO E14.5 NPCs. Each gene’s expression distribution is represented by a box (indicating the interquartile range), a horizontal line for the median, and whiskers extending to 1.5 times the interquartile range. Significance levels derived from differential expression analysis (DESeq2) are indicated by asterisks above each plot. **** *p* < 0.0001.

**
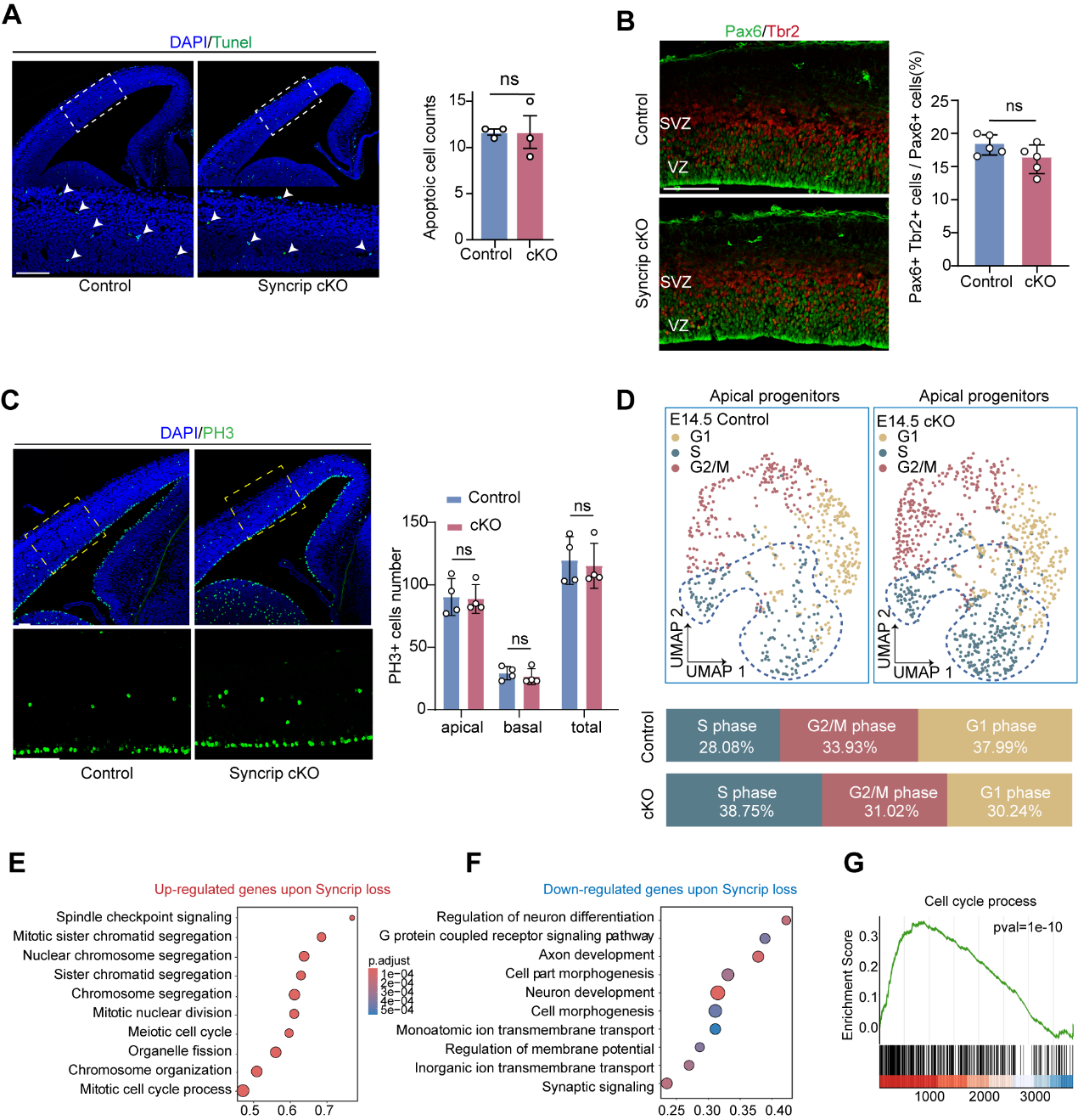
**

**Supplemental Figure S5. Syncrip Deletion Does Not Disrupt Apoptosis or NPCs to IPs Transition but Alters Cell Cycle Dynamics at E14.5.**

1. TUNEL^+^ cell staining in the E14.5 neocortex. The white boxes are enlarged at the bottom. Arrowheads indicate TUNEL^+^ cells.
2. Double staining of Pax6 and Tbr2 in coronal sections of the control (left) and Syncrip cKO (right) E14.5 neocortex. Quantification of the proportions of Pax6^+^ Tbr2^+^ cells and Pax6^+^ cells.
3. IF staining of PH3 in coronal sections of the control (left) and Syncrip cKO (right) E14.5 neocortex. Quantitative analysis of total PH3^+^ cell counts and their distribution in the apical and basal regions.
4. Cell cycle scoring and regression of Seurat were used to analyze the cell cycle of APs. Percentages of APs in different cell cycle phases.

(E-F) Gene Ontology (GO) analysis of upregulated and downregulated genes via RNA-seq of Syncrip cKO NPCs.

(G) GSEA revealed that the expression of “cell cycle progression” genes increased in Syncrip cKO NPCs.

Scale bar, 100 μm.

The data are presented as the mean ± SEM. Statistical significance was determined via an unpaired two-tailed Student’s *t* test.

**
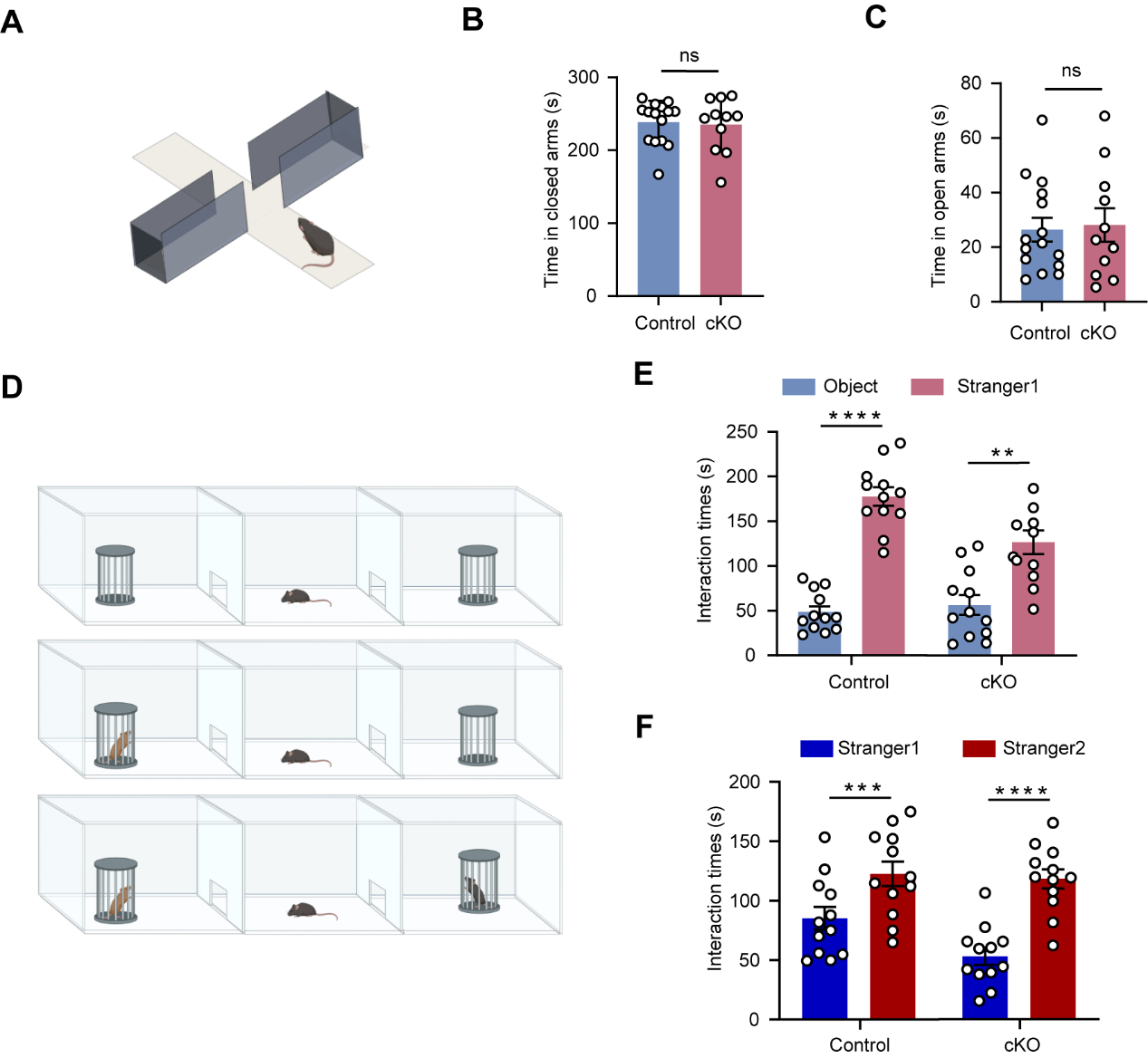
**

**Supplemental Figure S6. Syncrip Deletion does not Affect Anxiety or Social Interaction in Mice.**

1. Experimental scheme of the elevated plus maze.

(B-C) Quantification of time spent in the closed arms and in the open arms by control (n=15) and Syncrip cKO mice (n=11) in the elevated plus maze.

(D) Experimental scheme of the three-chamber test.

(E-F) Quantification of interaction times on different days by control (n=12) and Syncrip cKO mice (n=12).

The data are presented as the mean ± SEM. Statistical significance was determined via an unpaired two-tailed Student’s *t* test. ^**^*p* < 0.01, ^***^*p* < 0.001, ^****^*p*<0.0001


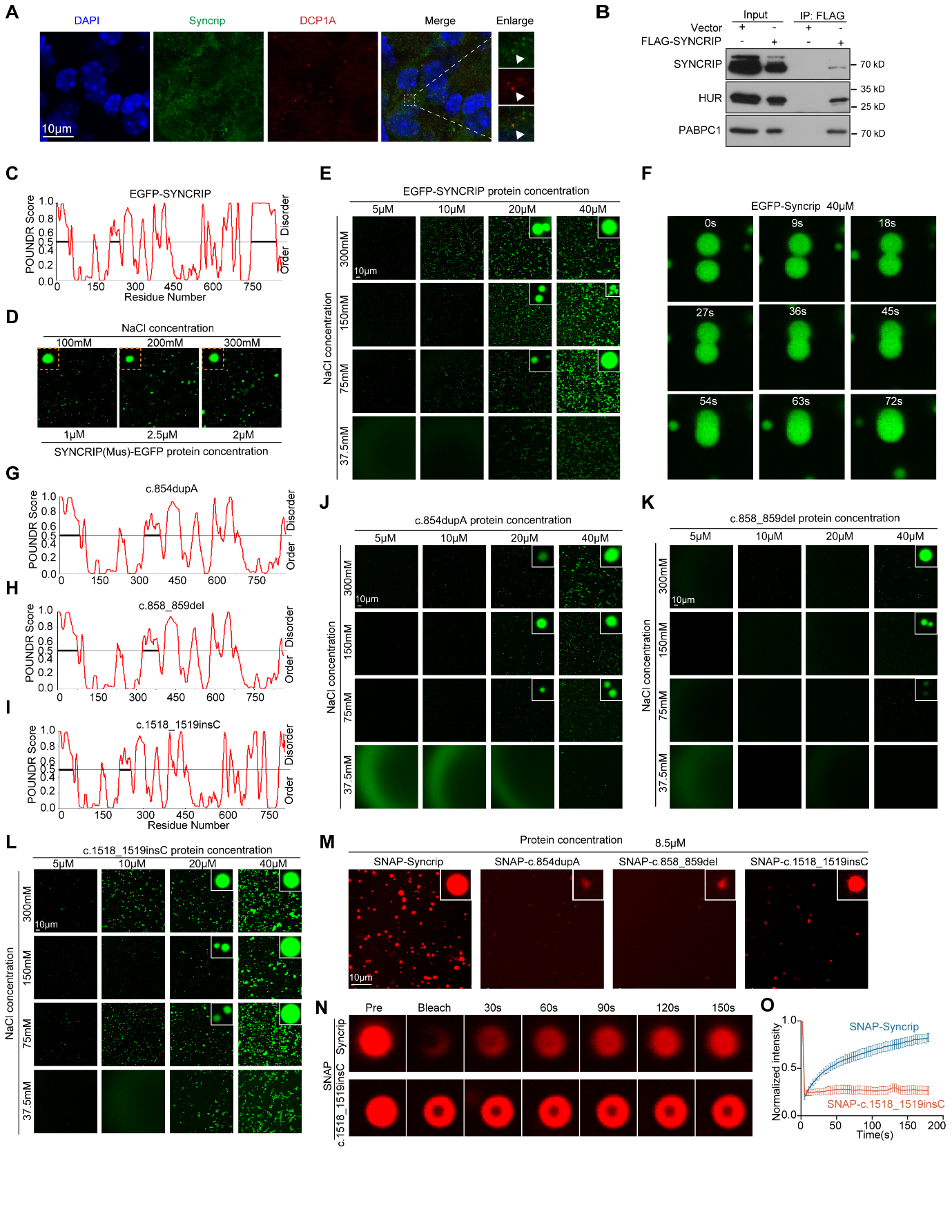


**Supplemental Figure S7. Analysis of Intrinsically Disordered Regions in SYNCRIP and the Phase Separation Properties of Disease-causing Mutants.**

1. Double staining of Syncrip and DCP1A in coronal sections of the P7 neocortex. Arrowheads indicate Syncrip^+^DCP1A^+^ cells.
2. Immunoblots showing protein interactions between SYNCRIP and HuR or PABPC1. Flag-Syncrip was transfected into HEK293T cells, and a co-IP assay was then performed. IP, immunoprecipitation; IB, immunoblot.
3. Predicted scoring of intrinsically disordered regions (IDRs) in the SYNCRIP protein
4. Phase plot of recombinant SYNCRIP(Mus)-EGFP at different protein and salt concentrations.
5. Phase plot of the recombinant EGFP-SYNCRIP protein at different protein and salt concentrations.
6. Representative images of two EGFP-SYNCRIP droplets undergoing fusion at 40 μM protein concentration.

(G-I) Predicted scoring of IDRs in the disease-causing mutants.

(J-L) Phase plot of EGFP-c.854dupA (J), EGFP-c.858_859del (K), and EGFP-c.1518_1519insC (L) mutants at different protein and salt concentrations.

(M) Phase separation assay using SNAP-tagged SYNCRIP and its mutants at 8.5 μM protein concentration.

(N-O) FRAP analysis of SNAP-SYNCRIP (n=9) and the SNAP-c.1518_1519insC (n=15). Representative images show fluorescence recovery after bleaching. Quantified fluorescence intensity recovery curves.

Scale bar, 10 μm


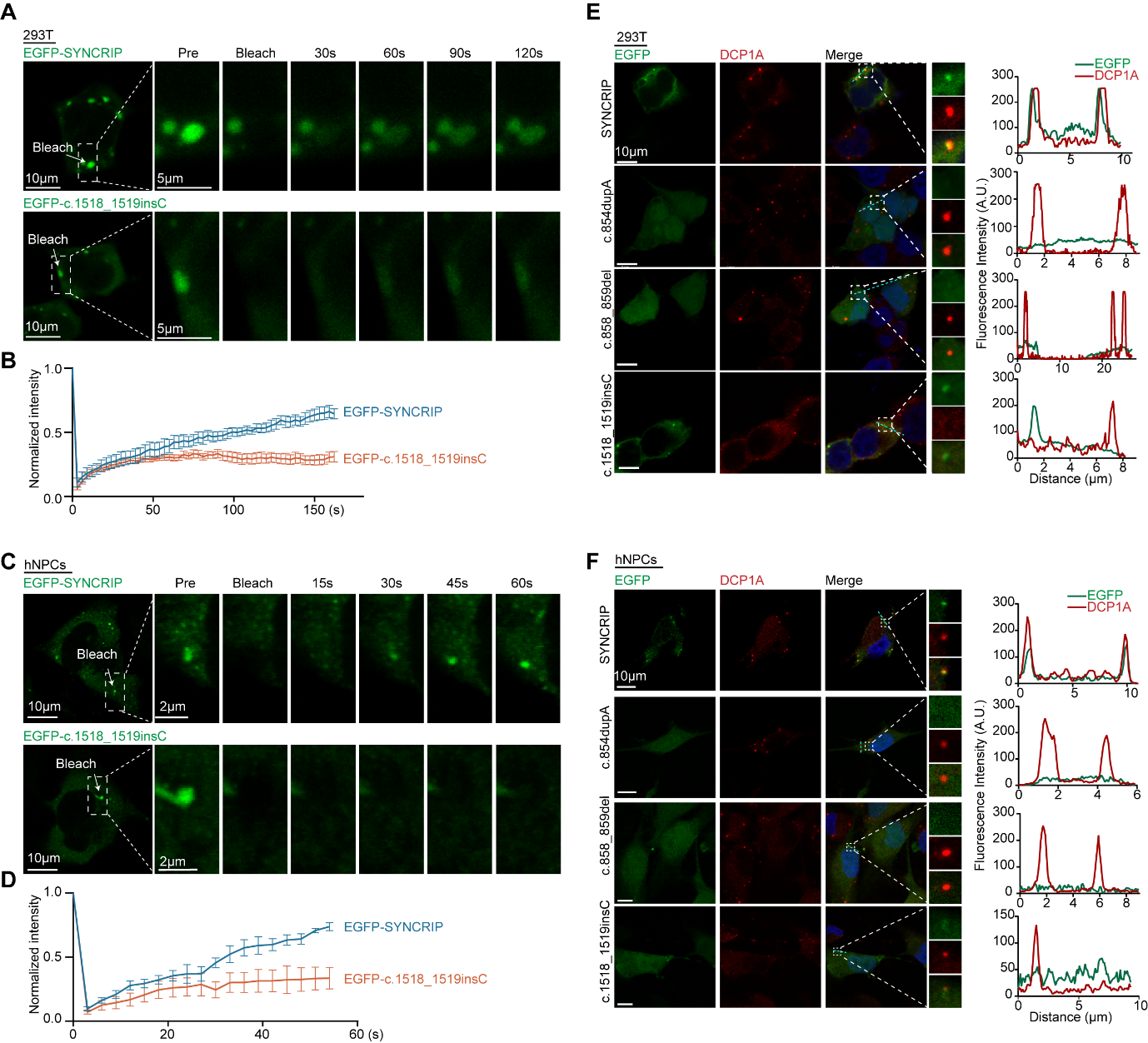


**Supplemental Figure S8. FRAP Analysis and Co-localization of Syncrip and its Pathogenic Mutants with DCP1A in 293T Cell Lines and Human Neural Progenitor (hNPCs) Cell Lines.**

(A-B) FRAP analysis in 293T cells. (A) Representative images of FRAP in EGFP-SYNCRIP and EGFP-c.1518_1519insC. Dashed boxes indicate the bleached regions, magnified in the inset. Scale bars: 10 μm (overview), 5 μm (magnified region). (B) Quantified fluorescence intensity recovery curves.

(C-D) FRAP analysis in hNPCs. (C) Representative images of FRAP in EGFP-SYNCRIP and EGFP-c.1518_1519insC. Dashed boxes indicate the bleached regions, magnified in the inset. Scale bars: 10 μm (overview), 2 μm (magnified region). (D) Quantified fluorescence intensity recovery curves.

(E-F) Co-localization analysis of EGFP-Syncrip and its pathogenic mutants (c.854dupA, c.858_859del, c.1518_1519insC) with DCP1A in 293T cells (E) and hNPCs (F). Representative images show EGFP (green), DCP1A (red), and merged channels with DAPI (blue). Dashed boxes indicate regions of interest, magnified in the inset. Scale bars, 10 μm. Fluorescence intensity profiles along the indicated lines.
